# Supplementary material for: Protein:Protein interactions in the cytoplasmic membrane apparently influencing sugar transport and phosphorylation activities of the e. coli phosphotransferase system
Source: PLoS One. 2019 Nov 21;14(11):e0219332. doi: 10.1371/journal.pone.0219332 (PMC6872149; doi:10.1371/journal.pone.0219332)
Supplement: S1 Table — E. coli WT was grown in LB or LB plus 0.2% fructose with 5 mM MgSO4 in both media. All [14C]substrates were used at 20 μM, final concentration, each of 5 μCi/μmole except for [3H]galactitol which was used at 30 μCi/μmole (see Experimental Procedures). Many values reported here and in subsequent tables have been rounded off. (DOCX) [file pone.0219332.s001.docx]

**S1 Table.** Effect of induction with fructose on the uptake of radioactive compounds as indicated below by the wild type *E. coli* strain BW25113 (WT). *E. coli* WT was grown in LB or LB plus 0.2% fructose with 5 mM MgSO_4_ in both media. All [^14^C]substrates were used at 20 μM, final concentration, each of 5 μCi/μmole except for [^3^H]galactitol which was used at 30 μCi/μmole (see Experimental Procedures). Many values reported here and in subsequent tables have been rounded off.

| **Radioactive substrate** | **Transport activity**  **(CPM/min/0.1 OD/0.1 ml)** | | | | **Relative transport activity**  **WT**  **(LB+Fructose /LB)** | | |
| --- | --- | --- | --- | --- | --- | --- | --- |
|  | **WT**  **(LB)** | | **WT**  **(LB+Fructose)** | |  |  |  |
|  | **Value** | **SD** | **Value** | **SD** | **Value** | **Average** | **SD** |
| **Fructose** | 30 | 2.3 | 330 | 10.1 | 11 | 11.0 | 0.2 |
|  | 29 | 1.9 | 314 | 1.2 | 11 |  |  |
| **Mannitol** | 56 | 0.6 | 416 | 53.9 | 7 | 7.0 | 0.6 |
|  | 53 | 1.7 | 349 | 8.3 | 7 |  |  |
| **N-acetylglucosamine** | 43 | 0.9 | 272 | 22.1 | 6 | 6.4 | 0.2 |
|  | 37 | 0.6 | 239 | 17.1 | 7 |  |  |
| **Methyl alpha** | 7 | 0.2 | 19 | 3.5 | 3 | 2.8 | 0.1 |
| **glucoside** | 7 | 0.6 | 19 | 0.5 | 3 |  |  |
| **2-Deoxyglucose** | 4 | 1.4 | 33 | 0.6 | 9 | 8.7 | 0.8 |
|  | 4 | 0.3 | 31 | 1.1 | 8 |  |  |
| **Trehalose** | 20 | 0.5 | 13 | 0.0 | 1 | 0.6 | 0.1 |
|  | 20 | 1.1 | 11 | 0.0 | 1 |  |  |
| **Galactitol** | 30 | 0.9 | 18 | 0.9 | 1 | 0.6 | 0.04 |
|  | 33 | 0.1 | 18 | 0.4 | 1 |  |  |
| **Galactose** | 17 | 0.6 | 22 | 1.7 | 1 | 1.3 | 0.03 |
|  | 17 | 0.5 | 22 | 0.8 | 1 |  |  |
