# Supplementary material for: Protein:Protein interactions in the cytoplasmic membrane apparently influencing sugar transport and phosphorylation activities of the e. coli phosphotransferase system
Source: PLoS One. 2019 Nov 21;14(11):e0219332. doi: 10.1371/journal.pone.0219332 (PMC6872149; doi:10.1371/journal.pone.0219332)
Supplement: S2 Table — The E. coli TM was grown in LB or LB plus 0.2% fructose with 5 mM MgSO4 in both media. All radioactive substrates were used at 20 μM, each containing 5 μCi/μmole 14C except for [3H]galactitol which was used at 30 μCi/μmole. These concentrations and specific activities were used throughout these studies for transport assays (see Experimental Procedures). (DOCX) [file pone.0219332.s002.docx]

**S2 Table.** Effect of growth with fructose on the uptake of radioactive substrates as indicated below by the triple mutant *E. coli* strain BW25113-*fruBKA:kn* (TM). The *E. coli* TM was grown in LB or LB plus 0.2% fructose with 5 mM MgSO_4_ in both media. All radioactive substrates were used at 20 μM, each containing 5 μCi/μmole ^14^C except for [^3^H]galactitol which was used at 30 μCi/μmole. These concentrations and specific activities were used throughout these studies for transport assays (see Experimental Procedures).

| **Radioactive substrate** | **Transport activity**  **(CPM/min/0.1 OD/0.1 ml)** | | | | **Relative transport activity**  **TM**  **(LB+Fructose / LB)** | | |
| --- | --- | --- | --- | --- | --- | --- | --- |
|  | **TM**  **(LB)** | | **TM**  **(LB+Fructose)** | |  |  |  |
|  | **Value** | **SD** | **Value** | **SD** | **Value** | **Average** | **SD** |
| **Fructose** | 13 | 3.5 | 11 | 2.8 | 0.9 | 0.9 | 0.02 |
|  | 13 | 4.0 | 11 | 1.8 | 0.8 |  |  |
| **Mannitol** | 78 | 3.2 | 120 | 1.4 | 1.5 | 1.5 | 0.05 |
|  | 72 | 0.9 | 105 | 0.6 | 1.5 |  |  |
| **N-Acetylglucosamine** | 56 | 3.9 | 85 | 8.3 | 1.5 | 1.5 | 0.04 |
|  | 52 | 1.7 | 81 | 4.1 | 1.6 |  |  |
| **Methyl alpha** | 6 | 0.2 | 7 | 0.2 | 1.1 | 1.2 | 0.11 |
| **glucoside** | 6 | 0.1 | 7 | 0.1 | 1.3 |  |  |
| **2-Deoxyglucose** | 4 | 0.5 | 16 | 1.5 | 3.7 | 3.4 | 0.41 |
|  | 5 | 0.3 | 15 | 1.1 | 3.1 |  |  |
| **Trehalose** | 19 | 0.8 | 30 | 1.4 | 1.5 | 1.5 | 0.02 |
|  | 19 | 1.1 | 29 | 0.8 | 1.5 |  |  |
| **Galactitol** | 37 | 3.2 | 62 | 1.3 | 1.7 | 1.7 | 0.12 |
|  | 35 | 0.4 | 65 | 0.6 | 1.8 |  |  |
| **Galactose** | 17 | 0.5 | 20 | 0.3 | 1.2 | 1.1 | 0.12 |
|  | 18 | 0.2 | 18 | 1.4 | 1.0 |  |  |
