# Supplementary material for: Protein:Protein interactions in the cytoplasmic membrane apparently influencing sugar transport and phosphorylation activities of the e. coli phosphotransferase system
Source: PLoS One. 2019 Nov 21;14(11):e0219332. doi: 10.1371/journal.pone.0219332 (PMC6872149; doi:10.1371/journal.pone.0219332)
Supplement: S3 Table — A. Effect of growth with fructose on the uptake of [14C]substrates as indicated below except for [3H]galactitol by the triple mutant E. coli strain BW25113-fruBKA:kn (TM) as compared to the wild type strain BW25113 (WT). E. coli strains were grown in LB plus 0.2% fructose and 5 mM MgSO4. B. Effect of mutations in the fruBKA operon on the uptake of [14C]compounds by the triple mutant E. coli strain BW25113-fruBKA:kn (TM) as compared to the wild type strain BW25113 (WT), both grown in LB medium. (DOCX) [file pone.0219332.s003.docx]

**S3 Table.** **A.** Effect of growth with fructose on the uptake of [^14^C]substrates as indicated below except for [^3^H]galactitol by the triple mutant *E. coli* strain BW25113-*fruBKA:kn* (TM) as compared to the wild type strain BW25113 (WT). *E. coli* strains were grown in LB plus 0.2% fructose and 5 mM MgSO_4._

| **Radioactive substrate** | **Transport activity**  **(CPM/min/0.1 OD/0.1 ml)** | | | | **Relative transport activity**  **(TM/WT)** | | |
| --- | --- | --- | --- | --- | --- | --- | --- |
|  | **WT**  **(LB+Fructose)** | | **TM**  **(LB+Fructose)** | |  |  |  |
|  | **Value** | **SD** | **Value** | **SD** | **Value** | **Average** | **SD** |
| **Fructose** | 248 | 8.6 | 10 | 0.8 | 0.04 | 0.05 | 0.01 |
|  | 206 | 2.8 | 11 | 3.5 | 0.05 |  |  |
| **Mannitol** | 298 | 7.5 | 120 | 16.9 | 0.4 | 0.47 | 0.1 |
|  | 174 | 8.8 | 94 | 5.0 | 0.54 |  |  |
| **N-acetylglucosamine** | 214 | 20.4 | 82 | 0.5 | 0.38 | 0.44 | 0.08 |
|  | 143 | 11.9 | 71 | 1.2 | 0.49 |  |  |
| **Methyl alpha** | 17 | 2.1 | 9 | 1.0 | 0.55 | 0.62 | 0.11 |
| **glucoside** | 14 | 0.5 | 10 | 0.02 | 0.7 |  |  |
| **2-Deoxyglucose** | 23 | 1.0 | 20 | 3.1 | 0.87 | 0.9 | 0.05 |
|  | 20 | 0.3 | 19 | 0.1 | 0.93 |  |  |
| **Trehalose** | 8 | 1.5 | 31 | 4.5 | 3.98 | 4.21 | 0.32 |
|  | 7 | 0.3 | 29 | 5.5 | 4.44 |  |  |
| **Galactitol** | 9 | 1.1 | 84 | 8.1 | 9.53 | 8.64 | 1.25 |
|  | 10 | 1.3 | 77 | 5.7 | 7.76 |  |  |
| **Galactose** | 16 | 0.4 | 17 | 1.2 | 1.05 | 0.99 | 0.08 |
|  | 16 | 0.1 | 15 | 1.4 | 0.94 |  |  |

**S3 Table. B.** Effect of mutations in the *fruBKA* operon on the uptake of [^14^C]compounds by the triple mutant *E. coli* strain BW25113-*fruBKA:kn* (TM) as compared to the wild type strain BW25113 (WT), both grown in LB medium.

| **Radioactive substrate** | **Transport activity**  **(CPM/min/0.1 OD/0.1 ml)** | | **Relative transport activity**  **(TM/WT)** | | |
| --- | --- | --- | --- | --- | --- |
|  | **WT**  **(LB)** | **TM**  **(LB)** |  |  |  |
|  | **Value** | **Value** | **Value** | **Average** | **SD** |
| **Mannitol** | 142 | 153 | 1.08 | 1.15 | 0.1 |
|  | 123 | 150 | 1.22 |  |  |
| **N-acetylglucosamine** | 100 | 114 | 1.14 | 1.15 | 0.01 |
|  | 86 | 100 | 1.16 |  |  |
| **Methyl alpha** | 7 | 7 | 0.92 | 0.9 | 0.02 |
| **glucoside** | 7 | 6 | 0.89 |  |  |
| **2-Deoxyglucose** | 11 | 11 | 1.08 | 1.06 | 0.03 |
|  | 10 | 10 | 1.05 |  |  |
| **Trehalose** | 29 | 52 | 1.8 | 1.52 | 0.39 |
|  | 24 | 29 | 1.25 |  |  |
| **Galactitol** | 36 | 32 | 0.9 | 0.88 | 0.03 |
|  | 34 | 29 | 0.85 |  |  |
| **Galactose** | 24 | 25 | 1.02 | 0.93 | 0.14 |
|  | 24 | 20 | 0.83 |  |  |
