# Supplementary material for: Protein:Protein interactions in the cytoplasmic membrane apparently influencing sugar transport and phosphorylation activities of the e. coli phosphotransferase system
Source: PLoS One. 2019 Nov 21;14(11):e0219332. doi: 10.1371/journal.pone.0219332 (PMC6872149; doi:10.1371/journal.pone.0219332)
Supplement: S4 Table — A. Effect of deletion of fruA on the uptake of [14C]compounds by the E. coli strain BW25113ΔfruA (WTΔfruA) as compared to the wild type strain BW25113 (WT). E. coli strains were grown in LB/0.2% fructose/5 mM MgSO4. B. Effect of deletion of fruA on the uptake of [14C]compounds by the E. coli strain BW25113ΔfruA (WTΔfruA) as compared to the wild type strain BW25113 (WT), both grown in LB medium. (DOCX) [file pone.0219332.s004.docx]

**S4 Table.** **A.** Effect of deletion of *fruA* on the uptake of [^14^C]compounds by the *E. coli* strain BW25113**∆***fruA* (WT**∆***fruA*) as compared to the wild type strain BW25113 (WT). *E. coli* strains were grown in LB/0.2% fructose/5 mM MgSO_4_.

| **Radioactive substrate** | **Transport activity**  **(CPM/min/0.1 OD/0.1 ml)** | | | | **Relative transport activity**  **(WT∆*fruA*/WT)** | | |
| --- | --- | --- | --- | --- | --- | --- | --- |
|  | **WT**  **(LB+Fructose)** | | **WT∆*fruA***  **(LB+Fructose)** | |  | | |
|  | **Value** | **SD** | **Value** | **SD** | **Value** | **Average** | **SD** |
| **Mannitol** | 388 | 15.14 | 192 | 2.53 | 0.49 | 0.53 | 0.05 |
|  | 348 | 5.38 | 194 | 8.51 | 0.56 |  |  |
| **N-acetylglucosamine** | 324 | 29.18 | 190 | 0.1 | 0.59 | 0.6 | 0.02 |
|  | 237 | 20.45 | 147 | 15.3 | 0.62 |  |  |
| **Methyl alpha** | 25 | 0.55 | 10 | 0.12 | 0.4 | 0.46 | 0.08 |
| **glucoside** | 24 | 0.1 | 12 | 0.16 | 0.51 |  |  |
| **2-Deoxyglucose** | 37 | 0.66 | 26 | 1.25 | 0.71 | 0.77 | 0.09 |
|  | 30 | 0.57 | 25 | 0.33 | 0.83 |  |  |
| **Trehalose** | 9 | 0.38 | 40 | 1.76 | 4.7 | 5.24 | 0.76 |
|  | 6 | 0.06 | 34 | 2.37 | 5.78 |  |  |
| **Galactitol** | 6 | 0.64 | 107 | 3.1 | 18.66 | 22.45 | 5.36 |
|  | 4 | 0.38 | 94 | 3.55 | 26.23 |  |  |
| **Galactose** | 18 | 0 | 25 | 0.16 | 1.43 | 1.27 | 0.23 |
|  | 15 | 2.11 | 17 | 0.67 | 1.11 |  |  |

**S4 Table.** **B.** Effect of deletion of *fruA* on the uptake of [^14^C]compounds by the *E. coli* strain BW25113**∆***fruA* (WT**∆***fruA*) as compared to the wild type strain BW25113 (WT), both grown in LB medium.

| **Radioactive substrate** | **Transport activity**  **(CPM/min/0.1 OD/0.1 ml)** | | **Relative transport activity**  **(WT∆*fruA*/WT)** | | |
| --- | --- | --- | --- | --- | --- |
|  | **WT**  **(LB)** | **WT∆*fruA***  **(LB)** |  |  |  |
|  | Value | Value | Value | Average | SD |
| **Mannitol** | 188 | 188 | 1 | 1.04 | 0.06 |
|  | 171 | 186 | 1.09 |  |  |
| **N-acetylglucosamine** | 168 | 170 | 1.02 | 1.01 | 0.01 |
|  | 152 | 152 | 1 |  |  |
| **Methyl alpha** | 8 | 9 | 1.09 | 1.1 | 0.01 |
| **glucoside** | 8 | 9 | 1.1 |  |  |
| **2-Deoxyglucose** | 14 | 14 | 1.01 | 1.04 | 0.03 |
|  | 13 | 14 | 1.06 |  |  |
| **Trehalose** | 33 | 34 | 1.04 | 1.06 | 0.03 |
|  | 26 | 28 | 1.08 |  |  |
| **Galactitol** | 44 | 35 | 0.79 | 0.81 | 0.03 |
|  | 40 | 33 | 0.83 |  |  |
| **Galactose** | 31 | 29 | 0.95 | 0.94 | 0.02 |
|  | 30 | 28 | 0.92 |  |  |
