# Supplementary material for: Protein:Protein interactions in the cytoplasmic membrane apparently influencing sugar transport and phosphorylation activities of the e. coli phosphotransferase system
Source: PLoS One. 2019 Nov 21;14(11):e0219332. doi: 10.1371/journal.pone.0219332 (PMC6872149; doi:10.1371/journal.pone.0219332)
Supplement: S5 Table — A. Effect of deletion of fruB on the uptake of [14C]compounds by the E. coli strain BW25113ΔfruB (WTΔfruB) as compared to the wild type strain BW25113 (WT), both grown in LB medium plus 0.2% fructose/5 mM MgSO4. B. Effect of deletion of fruB on the uptake of [14C]compounds by the E. coli strain BW25113ΔfruB (WTΔfruB) as compared to the wild type strain BW25113 (WT), both grown in LB medium. (DOCX) [file pone.0219332.s005.docx]

**S5 Table. A.** Effect of deletion of *fruB* on the uptake of [^14^C]compounds by the *E. coli* strain BW25113**∆***fruB* (WT**∆***fruB*) as compared to the wild type strain BW25113 (WT), both grown in LB medium plus 0.2% fructose/5 mM MgSO_4._

| **Radioactive substrate** | **Transport activity**  **(CPM/min/0.1 OD/0.1 ml)** | | | | **Relative transport activity**  **(WT∆*fruB*/WT)** | | |
| --- | --- | --- | --- | --- | --- | --- | --- |
|  | **WT**  **(LB+Fructose)** | | **WT∆*fruB***  **(LB+Fructose)** | |  |  |  |
|  | **Value** | **SD** | **Value** | **SD** | **Value** | **Average** | **SD** |
| **Mannitol** | 388 | 0.7 | 211 | 8.5 | 0.54 | 0.56 | 0.02 |
|  | 361 | 22.7 | 207 | 0.5 | 0.57 |  |  |
| **N-Acetylglucosamine** | 297 | 38.1 | 190 | 12.7 | 0.64 | 0.6 | 0.06 |
|  | 289 | 19.0 | 160 | 13.2 | 0.55 |  |  |
| **Methyl alpha** | 25 | 0.6 | 7 | 0.1 | 0.28 | 0.3 | 0.02 |
| **glucoside** | 25 | 0.5 | 8 | 0.3 | 0.31 |  |  |
| **2-Deoxyglucose** | 35 | 0.8 | 28 | 0.8 | 0.8 | 0.78 | 0.02 |
|  | 35 | 1.5 | 27 | 1.9 | 0.77 |  |  |
| **Trehalose** | 7 | 0.7 | 33 | 2.3 | 5.03 | 6.55 | 2.16 |
|  | 5 | 0.7 | 38 | 0.3 | 8.08 |  |  |
| **Galactitol** | 4 | 0.3 | 103 | 1.0 | 23.99 | 23.25 | 1.05 |
|  | 4 | 0.5 | 100 | 1.3 | 22.51 |  |  |
| **Galactose** | 16 | 1.2 | 22 | 0.6 | 1.36 | 1.42 | 0.09 |
|  | 17 | 0.2 | 25 | 2.6 | 1.49 |  |  |

**S5 Table. B.** Effect of deletion of *fruB* on the uptake of [^14^C]compounds by the *E. coli* strain BW25113**∆***fruB* (WT**∆***fruB*) as compared to the wild type strain BW25113 (WT), both grown in LB medium.

| **Radioactive substrate** | **Transport activity**  **(CPM/min/0.1 OD/0.1 ml)** | | **Relative transport activity**  **(WT∆*fruB*/WT)** | | |
| --- | --- | --- | --- | --- | --- |
|  | **WT**  **(LB)** | **WT∆*fruB***  **(LB)** |  |  |  |
|  | **Value** | **Value** | **Value** | **Average** | **SD** |
| **Mannitol** | 107 | 152 | 1.4 | 1.36 | 0.09 |
|  | 106 | 137 | 1.3 |  |  |
| **N-acetylglucosamine** | 90 | 108 | 1.2 | 1.23 | 0.03 |
|  | 81 | 101 | 1.3 |  |  |
| **Methyl alpha** | 7.5 | 6.8 | 0.9 | 0.91 | 0.01 |
| **glucoside** | 7.2 | 6.7 | 0.9 |  |  |
| **2-Deoxyglucose** | 12 | 13 | 1.1 | 1.18 | 0.1 |
|  | 9 | 11 | 1.3 |  |  |
| **Trehalose** | 25.5 | 29.6 | 1.2 | 1.27 | 0.15 |
|  | 18.4 | 25.4 | 1.4 |  |  |
| **Galactitol** | 46 | 28 | 0.6 | 0.71 | 0.14 |
|  | 33 | 26 | 0.8 |  |  |
| **Galactose** | 23.2 | 22.5 | 1.0 | 1.04 | 0.1 |
|  | 20.2 | 22.4 | 1.1 |  |  |
