# Supplementary material for: Protein:Protein interactions in the cytoplasmic membrane apparently influencing sugar transport and phosphorylation activities of the e. coli phosphotransferase system
Source: PLoS One. 2019 Nov 21;14(11):e0219332. doi: 10.1371/journal.pone.0219332 (PMC6872149; doi:10.1371/journal.pone.0219332)
Supplement: S6 Table — (DOCX) [file pone.0219332.s006.docx]

**S6 Table.** Effect of overexpression of *fruA* on the uptake of [^14^C]compounds by the recombinant triple mutant *E. coli* strain BW25113-*fruBKA:kn*-pMAL-FruA (TM-pMAL-*fruA*) as compared to the BW25113-*fruBKA:kn*-pML (TM-pMAL) strain.

| **Radioactive substrate** | **Transport activity**  **(CPM/min/0.1 OD/0.1 ml)** | | **Relative transport activity**  **(TM-pMAL-*fruA*/TM-pMAL)** | | |
| --- | --- | --- | --- | --- | --- |
|  | **TM-pMAL** | **TM-pMAL-*fruA*** |  |  |  |
|  | **Value** | **Value** | **Value** | **Average** | **SD** |
| **Mannitol** | 25 | 118 | 4.8 | 4.2 | 0.6 |
|  | 29 | 102 | 3.6 |  |  |
|  | 25 | 107 | 4.3 |  |  |
| **N-Acetylglucosamine** | 23 | 98 | 4.3 | 3.9 | 0.31 |
|  | 33 | 120 | 3.6 |  |  |
|  | 22 | 87 | 3.9 |  |  |
| **Methyl alpha glucoside** | 3 | 21 | 6.0 | 6.3 | 0.43 |
|  | 2 | 13 | 6.6 |  |  |
| **2-Deoxyglucose** | 2 | 21 | 12.3 | 11.2 | 1.55 |
|  | 2 | 16 | 10.1 |  |  |
| **Trehalose** | 12 | 19 | 1.6 | 1.6 | 0.07 |
|  | 13 | 20 | 1.6 |  |  |
|  | 12 | 18 | 1.5 |  |  |
| **Galactitol** | 19 | 54 | 2.9 | 2.4 | 0.42 |
|  | 23 | 54 | 2.4 |  |  |
|  | 18 | 37 | 2.0 |  |  |
| **Galactose** | 13 | 17 | 1.3 | 1.3 | 0.06 |
|  | 14 | 19 | 1.4 |  |  |
|  | 15 | 19 | 1.3 |  |  |

All stock radioactive substrates were used at 1 mM, each containing 5 μCi/μmole except for [3H] galactitol which was used at 30 μCi/μmole.
