# Supplementary material for: Protein:Protein interactions in the cytoplasmic membrane apparently influencing sugar transport and phosphorylation activities of the e. coli phosphotransferase system
Source: PLoS One. 2019 Nov 21;14(11):e0219332. doi: 10.1371/journal.pone.0219332 (PMC6872149; doi:10.1371/journal.pone.0219332)
Supplement: S7 Table — (DOCX) [file pone.0219332.s007.docx]

**S7 Table.** Effect of overexpression of *fruA* on the uptake of [^14^C]compounds by the recombinant *E. coli* strain BW25113-pMAL-*fruA* (WT-pMAL-*fruA*), as compared to the BW25113-pMAL (WT-pMAL) strain, both grown in LB medium.

| **Radioactive substrate** | **Transport activity**  **(CPM/min/0.1 OD/0.1 ml)** | | **Relative transport activity**  **(WT-pMAL-*fruA*/WT-pMAL)** | | |
| --- | --- | --- | --- | --- | --- |
|  | **WT-pMAL** | **WT-pMAL-*fruA*** |  |  |  |
|  |  |  | **Value** | **Average** | **SD** |
| **Fructose** | 15 | 42 | 2.8 | 2.9 | 0.19 |
|  | 13 | 39 | 3.0 |  |  |
| **Mannitol** | 25 | 124 | 4.9 | 4.7 | 0.3 |
|  | 26 | 119 | 4.5 |  |  |
| **N-Acetylglucosamine** | 23 | 82 | 3.6 | 3.6 | 0.05 |
|  | 24 | 86 | 3.5 |  |  |
| **Methyl alpha glucoside** | 4 | 18 | 4.5 | 4.8 | 0.43 |
|  | 4 | 18 | 5.1 |  |  |
| **2-Deoxyglucose** | 1 | 12 | 17.2 | 15.4 | 2.53 |
|  | 1 | 13 | 13.6 |  |  |
| **Trehalose** | 14 | 16 | 1.2 | 1.2 | 0.04 |
|  | 14 | 17 | 1.2 |  |  |
| **Galactitol** | 23 | 42 | 1.8 | 1.9 | 0.14 |
|  | 21 | 42 | 2.0 |  |  |
| **Galactose** | 18 | 17 | 0.9 | 1.0 | 0.03 |
|  | 16 | 16 | 1.0 |  |  |
