# Supplementary material for: Protein:Protein interactions in the cytoplasmic membrane apparently influencing sugar transport and phosphorylation activities of the e. coli phosphotransferase system
Source: PLoS One. 2019 Nov 21;14(11):e0219332. doi: 10.1371/journal.pone.0219332 (PMC6872149; doi:10.1371/journal.pone.0219332)
Supplement: S8 Table — (DOCX) [file pone.0219332.s008.docx]

**S8 Table.** Effect of overexpression of *fruB* on the uptake of [^14^C]compounds by the recombinant triple mutant *E. coli* strain BW25113-*fruBKA:kn*-pMAL-*fruB* (TM-pMAL-*fruA*) as compared to the BW25113-*fruBKA:kn*-pML (TM-pMAL) strain, both grown in LB medium.

| **Radioactive substrate** | **Transport activity**  **(CPM/min/0.1 OD/0.1 ml)** | | **Relative transport activity**  **(TM-pMAL-*fruB*/TM-pMAL)** | | |
| --- | --- | --- | --- | --- | --- |
|  | **TM-pMAL** | **TM-pMAL-*fruB*** |  |  |  |
|  | **Value** | **Value** | **Value** | **Average** | **SD** |
| **Mannitol** | 26 | 69 | 2.6 | 2.4 | 0.25 |
|  | 34 | 76 | 2.3 |  |  |
| **N-acetylglucosamine** | 29 | 65 | 2.3 | 2.2 | 0.14 |
|  | 34 | 72 | 2.1 |  |  |
| **Methyl alpha glucoside** | 4 | 5 | 1.4 | 1.3 | 0.08 |
|  | 4 | 5 | 1.3 |  |  |
| **2-Deoxyglucose** | 4 | 8 | 2.0 | 1.9 | 0.21 |
|  | 5 | 8 | 1.7 |  |  |
| **Trehalose** | 11 | 19 | 1.7 | 1.6 | 0.04 |
|  | 15 | 24 | 1.6 |  |  |
| **Galactitol** | 19 | 38 | 1.9 | 1.8 | 0.16 |
|  | 19 | 33 | 1.7 |  |  |
| **Galactose** | 13 | 16 | 1.3 | 1.5 | 0.27 |
|  | 13 | 21 | 1.6 |  |  |
