# Supplementary material for: Protein:Protein interactions in the cytoplasmic membrane apparently influencing sugar transport and phosphorylation activities of the e. coli phosphotransferase system
Source: PLoS One. 2019 Nov 21;14(11):e0219332. doi: 10.1371/journal.pone.0219332 (PMC6872149; doi:10.1371/journal.pone.0219332)
Supplement: S9 Table — (DOCX) [file pone.0219332.s009.docx]

**S9 Table.** Effect of overexpression of *fruB* on the uptake of [^14^C]compounds by the recombinant *E. coli* strain BW25113-pMAL-*fruB* (WT-pMAL-*fruB*), as compared to the BW25113-pMAL (WT-pMAL) strain, both grown in LB medium.

| **Radioactive substrate** | **Transport activity**  **(CPM/min/0.1 OD/0.1 ml)** | | **Relative transport activity**  **(WT-pMAL-*fruB*/WT-pMAL)** | | |
| --- | --- | --- | --- | --- | --- |
|  | **WT-pMAL** | **WT-pMAL-*fruB*** |  |  |  |
|  |  |  | **Value** | **Average** | **SD** |
| **Fructose** | 17 | 36 | 2.1 | 2.2 | 0.09 |
|  | 15 | 34 | 2.2 |  |  |
| **Mannitol** | 19 | 51 | 2.7 | 2.8 | 0.06 |
|  | 20 | 57 | 2.8 |  |  |
| **N-Acetylglucosamine** | 18 | 35 | 1.9 | 1.8 | 0.06 |
|  | 22 | 40 | 1.8 |  |  |
| **Methyl alpha glucoside** | 5 | 6 | 1.2 | 1.3 | 0.18 |
|  | 4 | 6 | 1.4 |  |  |
| **2-Deoxyglucose** | 1 | 5 | 4.2 | 4.4 | 0.38 |
|  | 1 | 6 | 4.7 |  |  |
| **Trehalose** | 11 | 15 | 1.5 | 1.5 | 0.09 |
|  | 11 | 18 | 1.6 |  |  |
| **Galactitol** | 22 | 31 | 1.5 | 1.5 | 0.01 |
|  | 21 | 30 | 1.4 |  |  |
| **Galactose** | 17 | 22 | 1.3 | 1.2 | 0.07 |
|  | 15 | 17 | 1.2 |  |  |
