# Supplementary material for: Protein:Protein interactions in the cytoplasmic membrane apparently influencing sugar transport and phosphorylation activities of the e. coli phosphotransferase system
Source: PLoS One. 2019 Nov 21;14(11):e0219332. doi: 10.1371/journal.pone.0219332 (PMC6872149; doi:10.1371/journal.pone.0219332)
Supplement: S10 Table — (DOCX) [file pone.0219332.s010.docx]

**S10 Table.** Effect of co-overexpression of *fruA* and *fruB* carried on two separate compatible plasmids on the uptake of [^14^C]compounds by the recombinant triple mutant *E. coli* strain BW25113-*fruBKA:kn*-pMAL-*fruA*-pZA31-*PtetM2*-*fruB* (TM-pMAL-*fruA*-pZA31-*PtetM2-fruB*) as compared to the BW25113-*fruBKA:kn*-pMAL-pZA31-*PtetM2-GFM* (TM-pMAL-pZA31-*PtetM2-GFM*) strain, both grown in LB medium.

| **Radioactive substrate** | **Transport activity**  **(CPM/min/0.1 OD/0.1 ml)** | | **Relative transport activity**  **(TM-pMAL-*fruA*/pZA31-PtetM2-*fruB*)/**  **(TM-pMAL/pZA31-*Ptet*M2-GFM)** | | |
| --- | --- | --- | --- | --- | --- |
|  | **TM-pMAL/pZA31-*Ptet*M2-GFM** | **TM-pMAL-*fruA*/pZA31-*Ptet*M2-*fruB*** |  | | |
|  | Value | Value | Value | Average | SD |
| **Fructose** | 10 | 105 | 10.9 | 10.4 | 0.59 |
|  | 12 | 124 | 10.0 |  |  |
| **Mannitol** | 32 | 143 | 4.4 | 4.5 | 0.13 |
|  | 32 | 149 | 4.6 |  |  |
| **N-Acetylglucos-amine** | 34 | 117 | 3.4 | 3.2 | 0.38 |
|  | 38 | 110 | 2.9 |  |  |
| **Methyl alpha glucoside** | 7 | 16 | 2.3 | 2.1 | 0.24 |
|  | 8 | 15 | 2.0 |  |  |
| **2-Deoxyglucose** | 2 | 21 | 10.5 | 9.8 | 0.98 |
|  | 2 | 20 | 9.1 |  |  |
| **Trehalose** | 17 | 50 | 2.9 | 2.7 | 0.32 |
|  | 19 | 48 | 2.5 |  |  |
| **Galactitol** | 27 | 98 | 3.6 | 3.3 | 0.47 |
|  | 35 | 103 | 3.0 |  |  |
| **Galactose** | 15 | 21 | 1.4 | 1.2 | 0.17 |
|  | 14 | 16 | 1.1 |  |  |
