# Supplementary material for: Protein:Protein interactions in the cytoplasmic membrane apparently influencing sugar transport and phosphorylation activities of the e. coli phosphotransferase system
Source: PLoS One. 2019 Nov 21;14(11):e0219332. doi: 10.1371/journal.pone.0219332 (PMC6872149; doi:10.1371/journal.pone.0219332)
Supplement: S11 Table — (DOCX) [file pone.0219332.s011.docx]

**S11 Table.** Effect of co-overexpression of *fruA* and *fruB* carried on two separate compatible plasmids on the uptake of [^14^C]compounds by the recombinant *E. coli* strain BW25113-pMAL-*fruA*-pZA31-*PtetM2-fruB* (WT-pMAL-*fruA*-pZA31-*PtetM2-fruB*) as compared to the BW25113-pMAL-pZA31-*PtetM2-GFM* (WT-pMAL-pZA31-*PtetM2-GFM*) strain, both grown in LB medium.

| **Radioactive substrate** | **Transport activity**  **(CPM/min/0.1 OD/0.1 ml)** | | **Relative transport activity**  **(WT-pMAL-*fruA*/pZA31-PtetM2-*fruB*)/**  **(WT-pMAL/pZA31-*Ptet*M2-GFM)** | | |
| --- | --- | --- | --- | --- | --- |
|  | **WT-pMAL/pZA31-**  ***Ptet*M2-GFM** | **WT-pMAL-*fruA*/pZA31-**  ***Ptet*M2-*fruB*** |  |  |  |
|  | **Value** | **Value** | **Value** | **Average** | **SD** |
| **Fructose** | 13 | 107 | 8.2 | 7.5 | 0.98 |
|  | 16 | 106 | 6.8 |  |  |
| **Mannitol** | 32 | 110 | 3.4 | 3.6 | 0.3 |
|  | 32 | 124 | 3.8 |  |  |
| **N-Acetylglucos-amine** | 33 | 66 | 2.0 | 2.0 | 0.08 |
|  | 35 | 66 | 1.9 |  |  |
| **Methyl alpha glucoside** | 9 | 17 | 2.0 | 2.1 | 0.14 |
|  | 9 | 19 | 2.2 |  |  |
| **2-Deoxyglucose** | 2 | 15 | 8.6 | 7.9 | 0.95 |
|  | 2 | 14 | 7.3 |  |  |
| **Trehalose** | 16 | 20 | 1.2 | 1.2 | 0.06 |
|  | 19 | 21 | 1.2 |  |  |
| **Galactitol** | 28 | 51 | 1.8 | 1.7 | 0.16 |
|  | 32 | 51 | 1.6 |  |  |
| **Galactose** | 12 | 11 | 0.9 | 0.9 | 0.1 |
|  | 13 | 10 | 0.8 |  |  |
