# Supplementary material for: Protein:Protein interactions in the cytoplasmic membrane apparently influencing sugar transport and phosphorylation activities of the e. coli phosphotransferase system
Source: PLoS One. 2019 Nov 21;14(11):e0219332. doi: 10.1371/journal.pone.0219332 (PMC6872149; doi:10.1371/journal.pone.0219332)
Supplement: S12 Table — (DOCX) [file pone.0219332.s012.docx]

**S12 Table.** Effect of overexpression of the *fruBKA* operon under *Ptet* promoter control on the uptake of [^14^C]compounds by the recombinant *E. coli* strain BW25113-*Chs kn:T:Ptet-fruBKA* (WT-*Ptet-fruBKA*) as compared to the BW25113 (WT) strain, both grown in LB medium.

| **Radioactive substrate** | **Transport activity**  **(CPM/min/0.1 OD/0.1 ml)** | | **Relative transport activity**  **(WT-*Ptet-fruBKA*/WT)** | | |
| --- | --- | --- | --- | --- | --- |
|  | **WT** | **WT-*Ptet-fruBKA*** |  |  |  |
|  | **Value** | **Value** | **Value** | **Average** | **SD** |
| **Fructose** | 51 | 198 | 3.9 | 4.3 | 0.62 |
|  | 54 | 254 | 4.7 |  |  |
| **Mannitol** | 125 | 238 | 1.9 | 2.1 | 0.21 |
|  | 103 | 227 | 2.2 |  |  |
| **N-acetylglucosamine** | 143 | 242 | 1.7 | 1.8 | 0.16 |
|  | 104 | 201 | 1.9 |  |  |
| **Methyl alpha glucoside** | 3 | 5 | 1.6 | 1.5 | 0.06 |
|  | 3 | 5 | 1.5 |  |  |
| **2-Deoxyglucose** | 4 | 10 | 2.6 | 2.6 | 0.06 |
|  | 3 | 8 | 2.5 |  |  |
| **Trehalose** | 29 | 32 | 1.1 | 1.1 | 0.02 |
|  | 26 | 29 | 1.1 |  |  |
| **Galactitol** | 26 | 39 | 1.5 | 1.5 | 0.09 |
|  | 29 | 40 | 1.4 |  |  |
| **Galactose** | 28 | 37 | 1.3 | 1.3 | 0.03 |
|  | 24 | 33 | 1.3 |  |  |
