# Supplementary material for: Protein:Protein interactions in the cytoplasmic membrane apparently influencing sugar transport and phosphorylation activities of the e. coli phosphotransferase system
Source: PLoS One. 2019 Nov 21;14(11):e0219332. doi: 10.1371/journal.pone.0219332 (PMC6872149; doi:10.1371/journal.pone.0219332)
Supplement: S13 Table — Values in the last column were calculated relative to the control without induction. A negative sign indicates a decrease in apparent expression level. All values are within experiment error. (DOCX) [file pone.0219332.s013.docx]

**S13 Table.** Effect of *fruBKA* operon induction with fructose on the expression of certain PTS transporters in *E. coli* using *lacZ* transcriptional fusions. Values in the last column were calculated relative to the control without induction. A negative sign indicates a decrease in apparent expression level. All values are within experiment error.

| ***E. coli* strain assayed** | **Growth conditions** | **Tested PTS transporter gene fused to *lacZ*** | **LacZ activity**  **(Miller units)**  **Value ± SD** | **% change**  **in expression** |
| --- | --- | --- | --- | --- |
| BW25113-*mtlA*-PZ | LB | *mtlA-PlacZ* | 115.13±1.66 |  |
| BW25113-*mtlA*-PZ | LB/Fructose | *mtlA-PlacZ* | 104.13±0.08 | -9.55 |
| BW25113-*manXYZ*-PZ | LB | *manXYZ-PlacZ* | 75.21±4.36 |  |
| BW25113-*manXYZ*-PZ | LB/Fructose | *manXYZ-PlacZ* | 51.44±2.23 | -31.6 |
| BW25113-*gatY*-PZ | LB | *gatY-PlacZ* | 4003.76±8.86 |  |
| BW25113-*gatY*-PZ | LB/Fructose | *gatY-PlacZ* | 1584 ± 8 | -60.43 |
| BW25113∆*fruBKA-mtlA*-PZ | LB | *mtlA-PlacZ* | 111.35±1.16 |  |
| BW25113∆*fruBKA-mtlA*-PZ | LB/Fructose | *mtlA-PlacZ* | 103.29±4.73 | -7.24 |
| BW25113∆*fruBKA-manXYZ*-PZ | LB | *manXYZ-PlacZ* | 100.04±2.33 |  |
| BW25113∆*fruBKA-manXYZ*-PZ | LB/Fructose | *manXYZ-PlacZ* | 157.53±5.19 | 57.47 |
| BW25113∆*fruBKA-gatY*-PZ | LB | *gatY-PlacZ* | 3841±44 |  |
| BW25113∆*fruBKA-gatY*-PZ | LB/Fructose | *gatY-PlacZ* | 4773±45 | 24.25 |
