# Supplementary material for: Protein:Protein interactions in the cytoplasmic membrane apparently influencing sugar transport and phosphorylation activities of the e. coli phosphotransferase system
Source: PLoS One. 2019 Nov 21;14(11):e0219332. doi: 10.1371/journal.pone.0219332 (PMC6872149; doi:10.1371/journal.pone.0219332)
Supplement: S14 Table — Values in the last column were calculated relative to the control without induction. A negative sign indicates a decrease in expression level. (DOCX) [file pone.0219332.s014.docx]

**S14 Table.** Effect of overexpression of the fructose transporter (FruA) gene (*fruA)* and the gene of its soluble partner FruB (*fruB)* on the expression of certain PTS transporters in *E. coli* using *lacZ* transcriptional fusions. Values in the last column were calculated relative to the control without induction. A negative sign indicates a decrease in expression level.

| ***E. coli* strain overexpressing a membrane transporter** | **Tested PTS transporter**  **fused to *lacZ*** | **LacZ activity**  **(Miller units)**  **Value ± SD** | **(%) change**  **in expression** |
| --- | --- | --- | --- |
| BW25113-*mtlA*-PZ-pMAL  (Wild type control strain) | *mtlA-PlacZ* | 125.81±0.42 |  |
| BW25113-*mtlA*-PZ-pMAL-*fruA*  (Wild type over expressing FruA) | *mtlA-PlacZ* | 150.63±0.69 | 19.7 |
| BW25113-*mtlA*-PZ-pMAL-*fruB*  (Wild type over expressing FruB) | *mtlA-PlacZ* | 141.67 ± 0.32 | 12.6 |
| BW25113-*manXYZ*-PZ-pMAL  (Wild type control strain) | *manXYZ-PlacZ* | 104.44 ± 2 |  |
| BW25113-*manXYZ*-PZ-pMAL-*fruA*  (Wild type over expressing FruA) | *manXYZ-PlacZ* | 136.64 ± 2.45 | 30.8 |
| BW25113-*manXYZ*-PZ-pMAL-*fruB*  (Wild type over expressing FruB) | *manXYZ-PlacZ* | 117.01 ± 0.62 | 12.0 |
| BW25113-*gatY*-PZ-pMAL  (Triple mutant control strain) | *gatY-PlacZ* | 3860.43 ± 137 |  |
| BW25113-*gatY*-PZ-pMAL-*fruA*  (Triple mutant over expressing FruA) | *gatY-PlacZ* | 3916.4 ± 163 | 1.4 |
| BW25113-*gatY*-PZ-pMAL-*fruB*  (Triple mutant over expressing FruB) | *gatY-PlacZ* | 4121.62 ± 62 | 6.8 |
| BW25113∆*fruBKA-mtlA*-PZ-pMAL  (Triple mutant control strain) | *mtlA-PlacZ* | 114.74 ± 2.14 |  |
| BW25113∆*fruBKA-mtlA*-PZ-pMAL-*fruA*  (Triple mutant over expressing FruA) | *mtlA-PlacZ* | 125.71 ± 0.73 | 9.6 |
| BW25113∆*fruBKA-mtlA*-PZ-pMAL-*fruB*  (Triple mutant over expressing FruB) | *mtlA-PlacZ* | 121.14 ± 1 | 5.6 |
| BW25113∆*fruBKA-manXYZ*-PZ-pMAL  (Triple mutant control strain) | *manXYZ-PlacZ* | 79.49 ± 0.1 |  |
| BW25113∆*fruBKA-manXYZ*-PZ-pMAL-*fruA*  (Triple mutant over expressing FruA) | *manXYZ-PlacZ* | 82.69 ± 1.4 | 4.0 |
| BW25113∆*fruBKA-manXYZ*-PZ-pMAL-*fruB*  (Triple mutant over expressing FruB) | *manXYZ-PlacZ* | 76.98 ± 0.34 | -3.2 |
| BW25113∆*fruBKA-gatY*-PZ-pMAL  (Triple mutant control strain) | *gatY-PlacZ* | 3873.2 ± 114 |  |
| BW25113∆*fruBKA-gatY*-PZ-pMAL-*fruA*  (Triple mutant over expressing FruA) | *gatY-PlacZ* | 4089.46 ± 99 | 5.6 |
| BW25113∆*fruBKA-gatY*-PZ-pMAL-*fruB*  (Triple mutant over expressing FruB) | *gatY-PlacZ* | 4171.21 ± 131 | 7.7 |
