# Supplementary material for: Protein:Protein interactions in the cytoplasmic membrane apparently influencing sugar transport and phosphorylation activities of the e. coli phosphotransferase system
Source: PLoS One. 2019 Nov 21;14(11):e0219332. doi: 10.1371/journal.pone.0219332 (PMC6872149; doi:10.1371/journal.pone.0219332)
Supplement: S15 Table — (DOCX) [file pone.0219332.s015.docx]

**S15 Table.** Effect of overexpression of some membrane transporter genes on the expression of other PTS transporters in *E. coli* using *lacZ* transcriptional fusions. Values in the last column were calculated relative to the control without induction. A negative sign indicates a decrease in expression level.

| **E. coli strain overexpressing membrane transporter** | **Tested PTS transporter**  **fused to *lacZ*** | **LacZ activity**  **(Miller units)**  **Value ± SD** | **% change in expression** |
| --- | --- | --- | --- |
| BW25113∆*fruBKA-mtlA*-PZ-pMAL/pZA31-*PtetM2-GFM*  (Triple mutant control strain) | *mtlA-PlacZ* | 155.08±6.34 |  |
| BW25113∆*fruBKA-mtlA*-PZ-pMAL-*fruA*/pZA31-*PtetM2-fruB*  (Triple mutant over expressing FruA and FruB) | *mtlA-PlacZ* | 158.76±0.6 | 2.4 |
| BW25113∆*fruBKA-manXYZ*-PZ-pMAL/pZA31-*PtetM2-GFM*  (Triple mutant control strain) | *manXYZ-PlacZ* | 138.74±3.51 |  |
| BW25113∆*fruBKA-manXYZ-*PZ-pMAL-*fruA*/pZA31-*PtetM2-fruB*  (Triple mutant over expressing FruA and FruB) | *manXYZ-PlacZ* | 158.35±1.14 | 14.1 |
| BW25113∆*fruBKA-gatY*-PZ-pMAL/pZA31-*PtetM2-GFM*  (Triple mutant control strain) | *gatY-PlacZ* | 4953.31±418 |  |
| BW25113∆fruBKA-gatY-PZ-pMAL-*fruA*/pZA31-*PtetM2-fruB*  (Triple mutant over expressing FruA and FruB) | *gatY-PlacZ* | 4310.56±514 | -13.0 |
