# Supplementary material for: Protein:Protein interactions in the cytoplasmic membrane apparently influencing sugar transport and phosphorylation activities of the e. coli phosphotransferase system
Source: PLoS One. 2019 Nov 21;14(11):e0219332. doi: 10.1371/journal.pone.0219332 (PMC6872149; doi:10.1371/journal.pone.0219332)
Supplement: S16 Table — Induction was conducted with 0.2% fructose in LB medium; ND, not determined. (DOCX) [file pone.0219332.s016.docx]

**S16 Table.** Effect of induction by fructose on PEP-dependent phosphorylation of some PTS sugars by the crude extract preparations of the wild type *E. coli* BW25113 (WT) and its triple mutant BW25113-*fruBKA:kn* (TM). Induction was conducted with 0.2% fructose in LB medium; ND, not determined.

| **PTS sugar** | **WT** | | | | | **TM** | | | | | **Relative Activity** | | | | | |
| --- | --- | --- | --- | --- | --- | --- | --- | --- | --- | --- | --- | --- | --- | --- | --- | --- |
|  | **LB** | **LB+ Fructose** | **Relative Activity**  **(LB+ Fructose /LB)** | | | **LB** | **LB+**  **Fructose** | **Relative Activity**  **(LB+ Fructose /LB)** | | | **TM/WT**  **(LB)** | | | **TM/WT**  **(LB+ Fructose)** | | |
|  | **CPM/μg** | **CPM/μg** | **Ratio** | **Average** | **SD** | **CPM/μg** | **CPM/μg** | **Ratio** | **Average** | **SD** | **Ratio** | **Average** | **SD** | **Ratio** | **Average** | **SD** |
| **Fructose** | 4 | 125 | 31.7 | 28.8 | 4.1 | ND | ND | ND | ND | ND | ND | ND | ND | ND | ND | ND |
|  | 4 | 113 | 25.9 |  |  | ND | ND | ND |  |  | ND |  |  | ND |  |  |
| **Mannitol** | 8 | 41 | 5.0 | 4.7 | 0.29 | 11 | 14 | 1.2 | 1.2 | 0.12 | 1.4 | 1.3 | 0.07 | 0.3 | 0.3 | 0.04 |
|  | 9 | 42 | 4.7 |  |  | 12 | 15 | 1.2 |  |  | 1.4 |  |  | 0.4 |  |  |
|  | 10 | 46 | 4.4 |  |  | 13 | 13 | 1.0 |  |  | 1.3 |  |  | 0.3 |  |  |
| **N-Acetylglu-cosamine** | 13 | 42 | 3.2 | 3.0 | 0.48 | 28 | 36 | 1.3 | 1.2 | 0.08 | 2.1 | 1.8 | 0.3 | 0.9 | 0.7 | 0.13 |
|  | 14 | 46 | 3.4 |  |  | 22 | 28 | 1.2 |  |  | 1.7 |  |  | 0.6 |  |  |
|  | 15 | 39 | 2.5 |  |  | 24 | 27 | 1.1 |  |  | 1.5 |  |  | 0.7 |  |  |
| **Methyl alpha glucoside** | 18 | 33 | 1.8 | 1.7 | 0.08 | 18 | 14 | 0.8 | 0.8 | 0.0 | 1.0 | 1.1 | 0.07 | 0.4 | 0.5 | 0.05 |
|  | 22 | 37 | 1.7 |  |  | 24 | 19 | 0.8 |  |  | 1.1 |  |  | 0.5 |  |  |
| **2-Deoxyglu-cose** | 10 | 16 | 1.6 | 1.6 | 0.08 | 19 | 23 | 1.2 | 1.1 | 0.09 | 1.9 | 1.8 | 0.2 | 1.5 | 1.3 | 0.3 |
|  | 10 | 17 | 1.7 |  |  | 16 | 17 | 1.1 |  |  | 1.6 |  |  | 1.0 |  |  |
| **Trehalose** | 27 | 25 | 1.0 | 1.0 | 0.03 | 28 | 25 | 0.9 | 0.9 | 0.02 | 1.0 | 1.2 | 0.19 | 1.0 | 1.1 | 0.18 |
|  | 19 | 19 | 1.0 |  |  | 25 | 23 | 0.9 |  |  | 1.3 |  |  | 1.2 |  |  |
| **Galactitol** | 4 | 1 | 0.2 | 0.1 | 0.04 | 7 | 31 | 4.4 | 3.9 | 0.59 | 1.7 | 1.4 | 0.33 | 43.4 | 39.9 | 4.94 |
|  | 5 | 1 | 0.1 |  |  | 6 | 22 | 3.5 |  |  | 1.2 |  |  | 36.4 |  |  |
