# Supplementary material for: Protein:Protein interactions in the cytoplasmic membrane apparently influencing sugar transport and phosphorylation activities of the e. coli phosphotransferase system
Source: PLoS One. 2019 Nov 21;14(11):e0219332. doi: 10.1371/journal.pone.0219332 (PMC6872149; doi:10.1371/journal.pone.0219332)
Supplement: S17 Table — (DOCX) [file pone.0219332.s017.docx]

**S17 Table.** Effect of overexpression of *fruA* on phosphorylation of fructose, mannitol, N-acetylglucosamine and galactitol by the membranous fractions of the wild type *E. coli* BW25113 strain (WT). A 30 μl aliquot of an 8 h HSS of the WT strain was used as a source of the soluble PTS enzymes.

| **PTS sugar** | **Specific phosphorylation activity**  **(CPM/ug)** | | | | | |
| --- | --- | --- | --- | --- | --- | --- |
|  | **WT-pMAL** | | **WT-pMAL-*fruA*** | | **Relative activity**  **(*fruA* OE/WT)** | |
|  | **Value** | **SD** | **Value** | **SD** | **Value** | **SD** |
| **Fructose** | 17 | 4 | 85 | 1 | 5.3 | 1.15 |
| **Mannitol** | 138 | 13 | 151 | 15 | 1.1 | 0.21 |
| **N-Acetylglucosamine** | 174 | 23 | 167 | 6 | 1.0 | 0.16 |
| **Galactitol** | 1493 | 66 | 451 | 1 | 0.3 | 0.02 |
