# Supplementary material for: Protein:Protein interactions in the cytoplasmic membrane apparently influencing sugar transport and phosphorylation activities of the e. coli phosphotransferase system
Source: PLoS One. 2019 Nov 21;14(11):e0219332. doi: 10.1371/journal.pone.0219332 (PMC6872149; doi:10.1371/journal.pone.0219332)
Supplement: S18 Table — (DOCX) [file pone.0219332.s018.docx]

**S18 Table.** Effect of overexpression of *fruA* or *fruB* on PEP-dependent phosphorylation of PTS sugars by crude extracts of the recombinant wild type *E. coli* strains BW25113-pMAL-*fruA* and BW25113-pMAL-*fruB* as compared to the control strain BW25113-pMAL.

| **PTS sugar** | **Specific activity (CPM/μg)** | | | **Relative activity** | | | | | |
| --- | --- | --- | --- | --- | --- | --- | --- | --- | --- |
|  | **WT-pMAL** | **WT-pMAL-*fruA*** | **WT-pMAL-*fruB*** | **OE *FruA*/WT** | | | **OE *FruB*/WT** | | |
|  |  |  |  | **Value** | **Average** | **SD** | **Value** | **Average** | **SD** |
| **Fructose** | 11 | 18 | 37 | 1.6 | 1.5 | 0.08 | 3.3 | 3.9 | 0.87 |
|  | 9 | 13 | 40 | 1.5 |  |  | 4.5 |  |  |
| **Mannitol** | 32 | 24 | 49 | 0.7 | 0.7 | 0.01 | 1.5 | 1.5 | 0.01 |
|  | 29 | 21 | 45 | 0.7 |  |  | 1.5 |  |  |
| **N-Acetylglucos-amine** | 55 | 40 | 66 | 0.7 | 0.7 | 0.01 | 1.2 | 1.1 | 0.12 |
|  | 52 | 39 | 54 | 0.7 |  |  | 1 |  |  |
| **Methyl alpha glucoside** | 50 | 56 | 72 | 1.1 | 1 | 0.12 | 1.4 | 1.3 | 0.14 |
|  | 65 | 61 | 81 | 0.9 |  |  | 1.3 |  |  |
| **2-Deoxyglucose** | 22 | 28 | 40 | 1.3 | 1.1 | 0.25 | 1.8 | 1.6 | 0.33 |
|  | 25 | 23 | 33 | 0.9 |  |  | 1.4 |  |  |
| **Trehalose** | 51 | 42 | 47 | 0.8 | 0.9 | 0.04 | 0.9 | 1.0 | 0.15 |
|  | 45 | 39 | 51 | 0.9 |  |  | 1.1 |  |  |
| **Galactitol** | 166 | 129 | 177 | 0.8 | 0.8 | 0.01 | 1.1 | 1.0 | 0.04 |
|  | 154 | 122 | 157 | 0.8 |  |  | 1 |  |  |
