# Supplementary material for: Protein:Protein interactions in the cytoplasmic membrane apparently influencing sugar transport and phosphorylation activities of the e. coli phosphotransferase system
Source: PLoS One. 2019 Nov 21;14(11):e0219332. doi: 10.1371/journal.pone.0219332 (PMC6872149; doi:10.1371/journal.pone.0219332)
Supplement: S19 Table — (DOCX) [file pone.0219332.s019.docx]

**S19 Table.** Effect of separate overexpression of *fruA* or *fruB* on the PEP-dependent phosphorylation of PTS sugars by the crude extracts of recombinant triple mutant *E. coli* strains BW25113-*fruBKA:kn*-pMAL-*fruA* and BW25113-*fruBKA:kn*-pMAL-*fruB* as compared to the control strain BW25113-*fruBKA:kn*-pMAL.

| **PTS sugar** | **Specific activity (CPM/μg)** | | | **Relative activity** | | | | | |
| --- | --- | --- | --- | --- | --- | --- | --- | --- | --- |
|  | **TM-pMAL** | **WT-pMAL-*fruA*** | **WT-pMAL-*fruB*** | **OE *fruA*/WT** | | | **OE *fruB*/WT** | | |
|  |  |  |  | **Value** | **Average** | **SD** | **Value** | **Average** | **SD** |
| **Fructose** | 3 | 3 | 5 | 1.0 | 1.0 | 0.02 | 1.4 | 1.5 | 0.12 |
|  | 3 | 3 | 4 | 1.0 |  |  | 1.6 |  |  |
| **Mannitol** | 59 | 46 | 56 | 0.8 | 0.7 | 0.11 | 0.9 | 0.9 | 0.05 |
|  | 62 | 39 | 54 | 0.6 |  |  | 0.9 |  |  |
| **N-acetylglucos-amine** | 51 | 43 | 53 | 0.8 | 0.9 | 0.08 | 1.0 | 1.0 | 0.04 |
|  | 49 | 46 | 48 | 1.0 |  |  | 1.0 |  |  |
| **Methy alpha glucoside** | 50 | 45 | 57 | 0.9 | 0.9 | 0.05 | 1.1 | 1.1 | 0.02 |
|  | 48 | 47 | 53 | 1.0 |  |  | 1.1 |  |  |
| **2-Deoxyglucose** | 31 | 25 | 33 | 0.8 | 0.8 | 0.01 | 1.1 | 1.0 | 0.05 |
|  | 28 | 23 | 28 | 0.8 |  |  | 1.0 |  |  |
| **Trehalose** | 47 | 41 | 51 | 0.9 | 0.9 | 0.07 | 1.1 | 1.1 | 0.03 |
|  | 44 | 43 | 49 | 1.0 |  |  | 1.1 |  |  |
| **Galactitol** | 80 | 69 | 70 | 0.9 | 1.0 | 0.17 | 0.9 | 0.9 | 0.09 |
|  | 74 | 81 | 74 | 1.1 |  |  | 1.0 |  |  |
