# Supplementary material for: Protein:Protein interactions in the cytoplasmic membrane apparently influencing sugar transport and phosphorylation activities of the e. coli phosphotransferase system
Source: PLoS One. 2019 Nov 21;14(11):e0219332. doi: 10.1371/journal.pone.0219332 (PMC6872149; doi:10.1371/journal.pone.0219332)
Supplement: S20 Table — (DOCX) [file pone.0219332.s020.docx]

**S20 Table.** Effect of simultaneous overexpression of *fruA* and *fruB* (carried on two separate compatible plasmids in the wild type *E. coli* BW25113 strain or its triple mutant BW25113-*fruBKA:kn*) on the PEP-dependent phosphorylation of some PTS sugars by crude extracts of strains BW25113-pMAL-pZA31-*PtetM2-GFM* (WT control), BW25113-pMAL-*fruA*-pZA31-*PtetM2*-*fruB* (WT O.E. *fruA-fruB*), BW25113-*fruBKA:kn*-pMAL-pZA31-*PtetM2*-*GFM* (TM-control) and BW25113-*fruBKA:kn*-pMAL-*fruA*-pZA31-*PtetM2*-*fruB* (TM-O.E *fruA.fruB*).

| **PTS sugar** | **Specific activity (CPM/μg)** | | **Relative activity** | | | **Specific activity (CPM/μg)** | | **Relative activity** | | |
| --- | --- | --- | --- | --- | --- | --- | --- | --- | --- | --- |
|  | **WT** | **WT OE-**  ***fruA-fruB*** | **WT OE-*fruA-fruB*/WT** | | | **TM** | **TM OE-**  ***fruA-fruB*** | **TM OE-*fruA-fruB*/TM** | | |
|  |  |  | **Ratio** | **Average** | **SD** |  |  | **Ratio** | **Average** | **SD** |
| **Fructose** | 6 | 421 | 69.5 | 75.5 | 6.5 | 5 | 456 | 87.9 | 85.5 | 4.3 |
|  | 6 | 409 | 71 |  |  | 5 | 394 | 85.8 |  |  |
|  | 6 | 520 | 83.6 |  |  | 7 | 574 | 88.8 |  |  |
|  | 6 | 490 | 77.8 |  |  | 8 | 598 | 79.3 |  |  |
| **Mannitol** | 20 | 306 | 15.4 | 14.8 | 2.9 | 34 | 351 | 10.2 | 11.2 | 2.8 |
|  | 21 | 256 | 12 |  |  | 33 | 304 | 9.3 |  |  |
|  | 18 | 334 | 18.7 |  |  | 26 | 401 | 15.4 |  |  |
|  | 24 | 324 | 13.3 |  |  | 33 | 331 | 10 |  |  |
| **N-Acetylglucos-amine** | 27 | 127 | 4.7 | 4.0 | 0.6 | 36 | 188 | 5.2 | 4.1 | 0.8 |
|  | 37 | 128 | 3.4 |  |  | 49 | 163 | 3.3 |  |  |
|  | 35 | 153 | 4.3 |  |  | 53 | 217 | 4.1 |  |  |
|  | 39 | 133 | 3.4 |  |  | 48 | 179 | 3.7 |  |  |
| **Methyl alpha glucoside** | 46 | 51 | 1.1 | 1.3 | 0.3 | 40 | 48 | 1.2 | 1.2 | 0.3 |
|  | 49 | 56 | 1.1 |  |  | 43 | 44 | 1 |  |  |
|  | 26 | 43 | 1.6 |  |  | 27 | 45 | 1.6 |  |  |
|  | 32 | 39 | 1.2 |  |  | 31 | 35 | 1.1 |  |  |
| **2-Deoxyglucose** | 14 | 28 | 2 | 1.8 | 0.2 | 25 | 50 | 2 | 1.8 | 0.1 |
|  | 15 | 26 | 1.8 |  |  | 28 | 49 | 1.7 |  |  |
|  | 17 | 29 | 1.7 |  |  | 30 | 57 | 1.9 |  |  |
|  | 14 | 24 | 1.7 |  |  | 27 | 47 | 1.7 |  |  |
| **Trehalose** | 27 | 48 | 1.8 | 1.4 | 0.3 | 33 | 74 | 2.3 | 1.7 | 0.5 |
|  | 29 | 45 | 1.6 |  |  | 36 | 70 | 2 |  |  |
|  | 27 | 33 | 1.2 |  |  | 31 | 45 | 1.5 |  |  |
|  | 28 | 30 | 1.1 |  |  | 34 | 43 | 1.3 |  |  |
| **Galactitol** | 168 | 92 | 0.5 | 0.6 | 0.1 | 225 | 108 | 0.5 | 0.5 | 0.1 |
|  | 171 | 107 | 0.6 |  |  | 187 | 105 | 0.6 |  |  |
|  | 84 | 55 | 0.7 |  |  | 148 | 68 | 0.5 |  |  |
|  | 87 | 56 | 0.6 |  |  | 135 | 62 | 0.5 |  |  |
