# Supplementary material for: Protein:Protein interactions in the cytoplasmic membrane apparently influencing sugar transport and phosphorylation activities of the e. coli phosphotransferase system
Source: PLoS One. 2019 Nov 21;14(11):e0219332. doi: 10.1371/journal.pone.0219332 (PMC6872149; doi:10.1371/journal.pone.0219332)
Supplement: S21 Table — (DOCX) [file pone.0219332.s021.docx]

**S21 Table.** Effect of overexpression of the *fruBKA* operon by changing its native promoter to *Ptet* on the PEP-dependent phosphorylation of mannitol, N-acetylglucosamine and galactitol by the membranous fraction of the wild type *E. coli* BW25113.

| **PTS sugar** | **Specific phosphorylation activity**  **(CPM/μg)** | | **Relative activity Ratio**  **(O.E *fruBKA*/WT)** | | |
| --- | --- | --- | --- | --- | --- |
|  | **BW25113 strain (WT)** | **BW25113-Chs *kn:T:Ptet-fruBKA* strain (O.E)** |  |  |  |
|  | **Value** | **Value** | **Value** | **Average** | **SD** |
| **Fructose** | 30 | 1217 | 40.0 | 43.3 | 4.69 |
|  | 24 | 1111 | 46.7 |  |  |
| **Mannitol** | 115 | 679 | 5.9 | 6.3 | 0.53 |
|  | 84 | 558 | 6.6 |  |  |
| **N-Acetylglucosamine** | 173 | 379 | 2.2 | 2.2 | 0.02 |
|  | 143 | 316 | 2.2 |  |  |
| **Galactitol** | 174 | 371 | 2.1 | 2.1 | 0.01 |
|  | 147 | 313 | 2.1 |  |  |
