# Supplementary material for: Protein:Protein interactions in the cytoplasmic membrane apparently influencing sugar transport and phosphorylation activities of the e. coli phosphotransferase system
Source: PLoS One. 2019 Nov 21;14(11):e0219332. doi: 10.1371/journal.pone.0219332 (PMC6872149; doi:10.1371/journal.pone.0219332)
Supplement: S22 Table — A crude extract of E. coli strain BW25113-mtlA:kn or BW25113ΔmtlA grown in LB + 0.2% fructose was prepared and used for testing the mannitol phosphorylation by FruA. (DOCX) [file pone.0219332.s022.docx]

**S22 Table.** Testing PEP-dependent phosphorylation of mannitol by a crude extract of an *E. coli* strain with an inactivated (BW25113-*mtlA:kn* strain) or deleted (BW25113∆*mtlA* strain) *mtlA* gene. A crude extract of *E. coli* strain BW25113-*mtlA:kn* or BW25113∆*mtlA* grown in LB + 0.2% fructose was prepared and used for testing the mannitol phosphorylation by FruA.

| **Strain applied** | **Relative activity**  **(BW25113 mutant/BW25113)** | |
| --- | --- | --- |
|  | **Value** | **SD** |
| **BW25113-*mtlA:kn*** | 0.02 | 0.01 |
| **BW25113∆*mtlA*** | 0.01 | 0 |
