# Supplementary material for: Protein:Protein interactions in the cytoplasmic membrane apparently influencing sugar transport and phosphorylation activities of the e. coli phosphotransferase system
Source: PLoS One. 2019 Nov 21;14(11):e0219332. doi: 10.1371/journal.pone.0219332 (PMC6872149; doi:10.1371/journal.pone.0219332)
Supplement: S23 Table — (DOCX) [file pone.0219332.s023.docx]

**S23 Table.** Effect of purified FruB on the PEP-dependent phosphorylation of PTS sugars by the crude extracts of recombinant triple mutant *E. coli* strain BW25113-*fruBKA*:*kn*-pMAL-*fruA* (Triple Mutant; TM-pMAL-*fruA*) and BW25113-*fruBKA*:*kn*-pMAL (TM-pMAL) strain.

| **PTS sugar** | | **Relative activity** | | | | | | | | | |
| --- | --- | --- | --- | --- | --- | --- | --- | --- | --- | --- | --- |
|  |  | **(EII prep plus purified FruB/EII prep alone) using purified FruB amount (μg) of:** | | | | | | | | | |
|  |  | **1.54** | | **3.07** | | **6.14** | | **9.21** | | **12.28** | |
|  |  | **TM-pMAL** | **TM-pMAL-*fruA*** | **TM-pMAL** | **TM-pMAL-*fruA*** | **TM-pMAL** | **TM-pMAL-*fruA*** | **TM-pMAL** | **TM-pMAL-*fruA*** | **TM-pMAL** | **TM-pMAL-*fruA*** |
| **Mannitol** | **value** | 1.8 | 9.1 | 2.4 | 10.9 | 3.7 | 14.3 | 4.6 | 16.9 | 5.9 | 18.1 |
|  | **SD** | 0.1 | 0.7 | 0.2 | 0.9 | 0.2 | 0.9 | 0.1 | 0.2 | 0.1 | 0.4 |
| **N-acetylglucos-amine** | **value** | 1.2 | 2.9 | 1.5 | 3.6 | 1.9 | 4.5 | 2.2 | 5.9 | 2.5 | 6.7 |
|  | **SD** | 0.1 | 0.2 | 0.2 | 0.4 | 0.3 | 0.6 | 0.2 | 0.5 | 0.1 | 0.2 |
| **Trehalose** | **value** |  | 1.5 |  | 1.4 |  | 1.9 |  |  |  |  |
|  | **SD** |  | 0.3 |  | 0.4 |  | 0.4 |  |  |  |  |
| **Methyl alpha glucoside** | **value** |  | 1.4 |  | 1.4 |  | 1.6 |  |  |  |  |
|  | **SD** |  | 0.3 |  | 0.3 |  | 0.4 |  |  |  |  |
| **2-Deoxyglucose** | **value** |  | 1.6 |  | 2.0 |  | 1.9 |  |  |  |  |
|  | **SD** |  | 0.4 |  | 0.5 |  | 0.5 |  |  |  |  |
| **Galactitol** | **value** |  | 1.1 |  | 1.1 |  | 1.2 |  |  |  |  |
|  | **SD** |  | 0.1 |  | 0.2 |  | 0.2 |  |  |  |  |
