# Supplementary material for: Protein:Protein interactions in the cytoplasmic membrane apparently influencing sugar transport and phosphorylation activities of the e. coli phosphotransferase system
Source: PLoS One. 2019 Nov 21;14(11):e0219332. doi: 10.1371/journal.pone.0219332 (PMC6872149; doi:10.1371/journal.pone.0219332)
Supplement: S24 Table — (DOCX) [file pone.0219332.s024.docx]

**S24 Table.** Effect of using a HSS of lysed *E. coli* BW25113 Chs *kn:T:Ptet-fruBKA* cells overexpressing the *fruBKA* operon on the PEP-dependent phosphorylation of fructose, mannitol, N-acetylglucosamine and galactitol by the wild type *E. coli* BW25113 overexpressing *fruA* or *galP.*

| **PTS sugar** | **Specific activity (CPM/μg)** | | | **Relative activity** | | | | | |
| --- | --- | --- | --- | --- | --- | --- | --- | --- | --- |
|  | **WT-**  **pMAL** | **WT-**  **pMAL-*fruA*** | **WT-**  **pMAL-*galP*** | **(OE *fruA* /WT)** | | | **(OE *galP*/WT)** | | |
|  |  |  |  | **Ratio** | **Average** | **SD** | **Ratio** | **Average** | **SD** |
| **Using 10 μl 8 h HSS of *E. coli* BW25113 chs *kn:T:Ptet-fruBKA*** | | | | | | | | | |
| **Fructose** | 54 | 1108 | 78 | 20 | 22.8 | 3.4 | 1.4 | 1.54 | 0.1 |
|  | 49 | 1244 | 81 | 25 |  |  | 1.6 |  |  |
| **Mannitol** | 76 | 720 | 105 | 9.5 | 9.1 | 0.5 | 1.4 | 1.29 | 0.1 |
|  | 53 | 466 | 65 | 8.8 |  |  | 1.2 |  |  |
| **N-Acetylglucos-amine** | 184 | 455 | 142 | 2.5 | 2.7 | 0.3 | 0.8 | 0.86 | 0.1 |
|  | 129 | 375 | 123 | 2.9 |  |  | 1.0 |  |  |
| **Galactitol** | 1669 | 377 | 302 | 0.2 | 0.2 | 0.0 | 0.2 | 0.19 | 0.0 |
|  | 1367 | 271 | 277 | 0.2 |  |  | 0.2 |  |  |
| **Using 30 μl 8 h HSS of *E. coli* BW25113 chs *kn:T:Ptet-fruBKA*** | | | | | | | | | |
| **Fructose** | 50 | 3030 | 113 | 60 | 55.7 | 6.9 | 2.3 | 2.04 | 0.3 |
|  | 57 | 2870 | 103 | 50 |  |  | 1.8 |  |  |
| **Mannitol** | 140 | 1104 | 151 | 7.9 | 8.4 | 0.7 | 1.7 | 1.1 | 0.0 |
|  | 184 | 1632 | 208 | 8.9 |  |  | 1.1 |  |  |
| **N-Acetylglucosamine** | 196 | 499 | 200 | 2.5 | 2.8 | 0.3 | 1.2 | 0.99 | 0.0 |
|  | 163 | 480 | 157 | 2.9 |  |  | 1.0 |  |  |
| **Galactitol** | 3242 | 668 | 591 | 0.2 | 0.2 | 0.0 | 0.2 | 0.2 | 0.0 |
|  | 3794 | 867 | 823 | 0.2 |  |  | 0.2 |  |  |
