# Supplementary material for: Protein:Protein interactions in the cytoplasmic membrane apparently influencing sugar transport and phosphorylation activities of the e. coli phosphotransferase system
Source: PLoS One. 2019 Nov 21;14(11):e0219332. doi: 10.1371/journal.pone.0219332 (PMC6872149; doi:10.1371/journal.pone.0219332)
Supplement: S25 Table — (DOCX) [file pone.0219332.s025.docx]

**S25 Table.** Effect of overexpression of *fruA* on transphosphorylation of PTS sugars by the membranous fraction of recombinant triple mutant *E. coli* strain BW25113-*fruBKA:kn*-pMAL-*fruA* as compared to control strain BW25113-*fruBKA:kn*.

| **PTS sugar** | **Specific Activity**  **(CPM/μg)** | | **Relative activity** | | |
| --- | --- | --- | --- | --- | --- |
|  | **TM-pMAL** | **TM-pMAL-*fruA*** | **OE *fruA*/TM** | | |
|  |  |  | **Value** | **Average** | **SD** |
| **Mannitol** | 77 | 67 | 0.9 | 1 | 0.14 |
|  | 49 | 52 | 1.1 |  |  |
|  | 59 | 67 | 1.1 |  |  |
| **N-Acetylglucos-amine** | 141 | 95 | 0.7 | 0.8 | 0.14 |
|  | 107 | 98 | 0.9 |  |  |
|  | 131 | 123 | 0.9 |  |  |
| **Methyl alpha glucoside** | 3960 | 4760 | 1.2 | 1.2 | 0.04 |
|  | 3890 | 4910 | 1.3 |  |  |
| **2-Deoxyglucose** | 2 | 2 | 1.2 | 1.2 | 0.01 |
|  | 2 | 2 | 1.2 |  |  |
| **Trehalose** | 2 | 1 | 0.5 | 0.5 | 0.02 |
|  | 2 | 1 | 0.5 |  |  |
