# Supplementary material for: Protein:Protein interactions in the cytoplasmic membrane apparently influencing sugar transport and phosphorylation activities of the e. coli phosphotransferase system
Source: PLoS One. 2019 Nov 21;14(11):e0219332. doi: 10.1371/journal.pone.0219332 (PMC6872149; doi:10.1371/journal.pone.0219332)
Supplement: S26 Table — (DOCX) [file pone.0219332.s026.docx]

**S26 Table.** Effect of purified FruB on transphosphorylation of PTS sugars by the recombinant triple mutant *E. coli* strain BW25113-*fruBKA*:kn-pMAL (TM-pMAL) and its *fruA* overexpressing strain BW25113-*fruBKA*:kn-pMAL-fruA (TM-pMAL-*fruA*).

| **Radioactive sugar** | **Relative enzyme activity (EII plus purified FruB /EII alone)** | | | | | | | | | |
| --- | --- | --- | --- | --- | --- | --- | --- | --- | --- | --- |
|  | **TM-pMAL-*fruA*** | | | | | | | | **TM-pMAL** | |
|  | **Purified FruB (μg)** | | | | | | | | **Purified FruB (μg)** | |
|  | **0.24** | | **0.37** | | **1.48** | | **1.85** | | **1.85** | |
|  | Value | SD | Value | SD | Value | SD | Value | SD | Value | SD |
| **Mannitol** | 1.01 | 0.1 | 0.98 | 0.11 | 0.87 | 0.05 | 0.8 | 0.08 | 1.07 | 0.13 |
| **N-Acetylglucos-amine** | 1.05 | 0.09 | 0.98 | 0.08 | 1.05 | 0.24 | 1.15 | 0.15 | 1.1 | 0.17 |
| **Trehalose** | 1.06 | 0.09 | 0.92 | 0.01 | 0.94 | 0.06 | 0.96 | 0.06 | 1.07 | 0.08 |
| **Methyl alpha glucoside** | 0.97 | 0.04 | 1.01 | 0.02 | 1.04 | 0.01 | 0.97 | 0.09 | 1.02 | 0.08 |
| **2-Deoxyglucose** | 1.13 | 0.13 | 1.12 | 0.2 | 1 | 0.05 | 1.02 | 0.16 | 1.03 | 0.13 |
