# Supplementary material for: Protein:Protein interactions in the cytoplasmic membrane apparently influencing sugar transport and phosphorylation activities of the e. coli phosphotransferase system
Source: PLoS One. 2019 Nov 21;14(11):e0219332. doi: 10.1371/journal.pone.0219332 (PMC6872149; doi:10.1371/journal.pone.0219332)
Supplement: S27 Table — (DOCX) [file pone.0219332.s027.docx]

# S27 Table. Strains and plasmids used in this study

| **Strains or plasmids** | **Genotype or description** | **Reference or source** |
| --- | --- | --- |
| **Strains** |  |  |
| BW25113 | Wild type, *lacI*^q^ *rrnB*_T14_ Δ*lacZ*_WJ_ Δ*hsdR*514 Δ*araBAD*_AH33_ Δ*rhaBAD*_LD78_ | Wanner (2000) |
| Δ*fruBKA* | Δ*fruBKA* in BW25113 | This study |
| Δ*mtlA* | Δ*mtlA* in BW25113 | This study |
| Δ*fruA* | Δ*fruA* in BW25113 | This study |
| Δ*fruB* | Δ*furB* in BW25113 | This study |
| BW_P*tet*-*fruBKA* | P*tet* driven *fruBKA* expression in BW25113 | This study |
| BW_P*mtlA*-*lacZ* | BW25113 in which P*mtlA* is fused to *lacZ* at the *lac* locus | This study |
| BW_P*man*-*lacZ* | BW25113 in which P*manXYZ* is fused to *lacZ* at the *lac* locus | This study |
| BW_P*gatY*-*lacZ* | BW25113 in which *wt* P*gatY* is fused to *lacZ* at the *lac* locus | This study |
| Δ*fruBKA*_P*mtlA*-*lacZ* | Δ*fruBKA* in which P*mtlA* is fused to *lacZ* at the *lac* locus | This study |
| Δ*fruBKA*_P*man*-*lacZ* | Δ*fruBKA* in which P*manXYZ* is fused to *lacZ* at the *lac* locus | This study |
| Δ*fruBKA*_P*gatY*-*lacZ* | Δ*fruBKA* in which P*gatY* is fused to *lacZ* at the *lac* locus | This study |
|  |  |  |
| **Plasmids** |  |  |
| pMAL | pMAL-p2x in which the *BglI*I/*BamH*I flanked region containing *malE* is deleted | This study |
| pMAL-*fruA* | Ptac driving *fruA* in pMAL | This study |
| pMAL-*fruB* | Ptac driving *fruB* in pMAL | This study |
| pMAL-*galP* | Ptac driving *galP* in pMAL | This study |
| pKDT | Insertion of an *rrnB* terminator downstream of km gene in pKD13 | Klumpp et al. (2009) |
| pKDT_P*tet* | P*tet* cloned downstream of the *rrnB* terminator in pKDT | Klumpp et al. (2009) |
| pKDT_P*mtlA* | P*mtlA* cloned downstream of the *rrnB* terminator in pKDT | This study |
| pKDT_P*man* | P*man* cloned downstream of the *rrnB* terminator in pKDT | This study |
| pKDT_P*gatY* | P*gatY*cloned downstream of the *rrnB* terminator in pKDT | This study |
| pUT18-*fruA* | *fruA* is transnationally fused to N-terminus of T18 domain in PUT18 | This study |
| pUT18-*gatC* | *gatC* is transnationally fused to N-terminus of T18 domain in pUT18 | This study |
| pUT18-*nagE* | *nagE* is transnationally fused to N-terminus of T18 domain in pUT18 | This study |
| pUT18-*treB* | *treB* is transnationally fused to N-terminus of T18 domain in pUT18 | This study |
| pUT18-*mtlA* | *mtlA* is transnationally fused to N-terminus of T18 domain in pUT18in pUT18 | This study |
| pUT18-*fruB* | *fruB* is transnationally fused to N-terminus of T18 domain in pUT18 | This study |
| pKNT25-*fruA*  pKNT25-*fruB* | *fruA* is transnationally fused to N-terminus of N25 domain in pKNT25  *fruB* is transnationally fused to N-terminus of N25 domain in pKNT25 | This study  This study |
| pKNT25-*mtlA*  pKNT25-*nagE* | *mtlA* is transnationally fused to N-terminus of N25 domain in pKNT25  *nagE* is transnationally fused to N-terminus of N25 domain in pKNT25 | This study  This study |
