# Supplementary material for: Protein:Protein interactions in the cytoplasmic membrane apparently influencing sugar transport and phosphorylation activities of the e. coli phosphotransferase system
Source: PLoS One. 2019 Nov 21;14(11):e0219332. doi: 10.1371/journal.pone.0219332 (PMC6872149; doi:10.1371/journal.pone.0219332)
Supplement: S28 Table — (DOCX) [file pone.0219332.s028.docx]

**S28 Table. Oligonucleotides used in this study**

| **Name** | **Sequence** | **Use** |
| --- | --- | --- |
| fruB1-P1 | cgaaaaggccggagacaaagaagaggcgattcgccaggtcgctgcggcgctgtgtaggctggagctgcttcg | Chromosomal *fruBKA* deletion |
| fruA2-P2 | gctaccaggtaacccaataccggcgtaatagcgccagggatcagcagaacatatgaatatcctccttagttc | Chromosomal *fruBKA* deletion |
| fruA-ver-R | attacgctgctttcgctactgcgtc | Verification of *fruBKA* deletion |
| mtlA1-P1 | tcggcgcgtttatcgcgtggggtatcatcaccgcgttatttattccaacatgtgtaggctggagctgcttcg | Chromosomal *mtlA* deletion |
| mtlA2-P2 | atgacggactcatcatccagtgcattggtcaggctggtgataacctgaatcatatgaatatcctccttagttc | Chromosomal *mtlA* deletion |
| mtlA-ver-R | accttctccatgtggagagggtg | Verification of *fruB* deletion |
| fruB1-P1 | catccgggcgaaaaggccggagacaaagaagaggcgattcgccaggtcgctgtgtaggctggagctgcttcg | Chromosomal *fruB* deletion |
| fruB2-P2 | cagcatctgcaccctgggcggtaaagcgtaggcgatgacctttcttaacgcatatgaatatcctccttagttc | Chromosomal *fruB* deletion |
| fruB-ver-R1 | gcagaaaccaacaaggtcataagc | Verification of *fruB* deletion |
| fruA1-P1 | tattgacgctaatctcggtcaggcacgcgcctatatggcgaagaccctgctgtgtaggctggagctgcttcg | Chromosomal *fruA* deletion |
| fruA2-P2 | tccacttccggacgtttcaggaaggcataggccaaacccgccaccagcgtcatatgaatatcctccttagttc | Chromosomal *fruA* deletion |
| fruA-ver-R1 | tctttgcgctcatcaaatgttacag | Verification of *fruA* deletion |
| PtetfruB-P1 | tgcagcacatcaaacttttgctcataactttacggctttccttgcgtgctgtgtaggctggagctgcttc | Replacing P*fruB* by Ptet |
| PtetfruB-P2 | gtctccggccttttcgcccggatggatgtcctgtacggataactggaacatggtacctttctcctctttaatgaattc | Replacing P*fruB* by Ptet |
| fruB-ver-R2 | tgtcggtagtgccgtgtggaatagc | Verification of Ptet-*fruBKA* |
| mtlAp-Xh-F | atactcgagacgattgtatgacgaaggcataacatg | Cloning P*mtlA* in pKDT |
| mtlAp-Bm-R | aatggatccaccatgttgctgaggaaacgaccaaag | Cloning P*mtlA* in pKDT |
| manXp-Xh-F | atactcgagaccttcctttgcaaacgaatgtgacaag | Cloning P*manX* in pKDT |
| manXp-Bm-R | ttaggatcctgccgttttaagcaactgctctgcag | Cloning P*manX* in pKDT |
| gatYp-Xh-F | atactcgagtgcctacatagcactgccacgtatg | Cloning P*gatY* in pKDT |
| gatYp-Bm-R | attggatccttagttgttcagcatctgctttgtcgatac | Cloning P*gatY* in pKDT |
| mtlAp-Z-P2 | cgacggccagtgaatccgtaatcatggtcatagctgtttcctgtgtgaaattacatgttgctgaggaaacgaccaaagc | P*mtlA*:*lacZ* transcriptional fusion |
| manXp-Z-P2 | cgacggccagtgaatccgtaatcatggtcatagctgtttcctgtgtgaaattaaagcaactgctctgcagcccaaccatg | P*manX*:*lacZ* transcriptional fusion |
| gatYp-Z-P2 | cgacggccagtgaatccgtaatcatggtcatagctgtttcctgtgtgaaattagttgttcagcatctgctttgtcg | P*gatY*:*lacZ* transcriptional fusion |
| fruA-Nd-F | atacatatgaaaacgctgctgattattgacgc | Cloning *fruA* into pMAL |
| fruAhis-Sal-R | aatgtcgacttagtgatgatgatgatgatgacctccacccgctgctttcgctactgcgtccacttc | Cloning *fruA* into pMAL |
| fruB-Nd-F | atacatatgttccagttatccgtacaggacatc | Cloning *fruB* into pMAL |
| fruBhis-Sal-R | aatgtcgacttagtgatgatgatgatgatgacctccacctgcgccctccccaagaccagcagcg | Cloning *fruB* into pMAL |
| galP-Nd-F | atacatatgcctgacgctaaaaaacaggggcggtc | Cloning *galP* into pMAL |
| galPhis-Sal-R | aatgtcgacttagtgatgatgatgatgatgacctccaccatcgtgagcgcctatttcgcgcag | Cloning *galP* into pMAL |
| pMAL-ver-F | tcgtataatgtgtggaattgtgagc | Verifying DNA cloning in pMAL |
| fruA-ver-F1 | ttgctggtaaaccgatgtatcgtac | Verifying *fruA* cloning in pMAL |
| fruA-ver-F2 | ctaatttccagtctggtggtcggtc | Verifying *fruA* cloning in pMAL |
| fruB-ver-F | gttgacgccactttcgtcaccaaag | Verifying *fruB* cloning in pMAL |
| galP-ver-F1 | tgatctcgatgtatcagttgatgatc | Verifying *galP* cloning in pMAL |
| galP-ver-F2 | tgaccaacgtacttgccacctttatcg | Verifying *galP* cloning in pMAL |
