# Supplementary material for: Protein:Protein interactions in the cytoplasmic membrane apparently influencing sugar transport and phosphorylation activities of the e. coli phosphotransferase system
Source: PLoS One. 2019 Nov 21;14(11):e0219332. doi: 10.1371/journal.pone.0219332 (PMC6872149; doi:10.1371/journal.pone.0219332)
Supplement: S1 Fig — pKNT25-fruA (expression in low copy plasmid pKNT25) against pUT18-gatC, pUT18-nagE, pUT18-treB, pUT18-mtlA, and pUT18-fruB (expression in high copy plasmid pUT18); and pUT18-fruB against pKNT25-mtlA and pKNT25-nagE are presented. As reported by Babu et al., 2018, FruA interacts with other membrane proteins. In this study, the interactions of FruA could be confirmed for GatC, NagE, TreB and MtlA, and the interactions of FruB could be detected and reproduced for MtlA and NagE. The interactions of FruB, the soluble partner, with FruA can be considered as a positive control. Note that the interactions of FruB with MtlA and NagE appear to be substantially weaker than those with FruA. (DOCX) [file pone.0219332.s029.docx]

| Control | 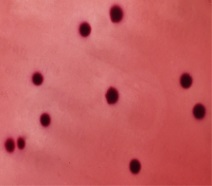  Positive control | 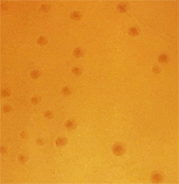  pKNT25-*fruA*#pUT18-*zip* (as negative control) |  |  |  |
| --- | --- | --- | --- | --- | --- |
| FruA interactios | 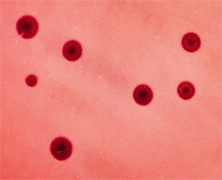  pKNT25-*fruA*  # pUT18-*gatC* | 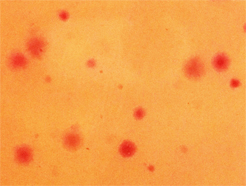  pKNT25-*fruA*  # pUT18-*nagE* | 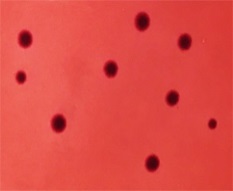  pKNT25-*fruA*  # pUT18-*treB* | 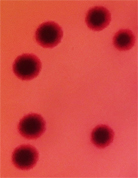  pKNT25-*fruA* # pUT18-*mtlA* | 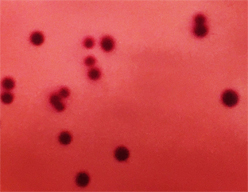  pKNT25-*fruA*#pUT18-*fruB* |
| FruB interactions | 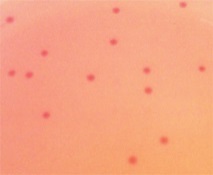  pUT18-*fruB* # pKNT25-*mtlA* | 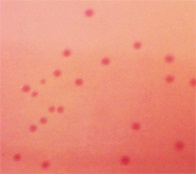  pUT18-*fruB* # pKNT25-*nagE* |  |  |  |

**S1 Fig.** Results from the use of a bacterial two hybrid system for testing interactions of FruA and FruB with other membrane proteins. pKNT25-*fruA* (expression in low copy plasmid pKNT25) against pUT18-*gatC*, pUT18-*nagE*, pUT18-*treB*, pUT18-*mtlA*, and pUT18-*fruB* (expression in high copy plasmid pUT18); and pUT18-*fruB* against pKNT25-*mtlA* and pKNT25-*nagE* are presented. As reported by Babu et al., 2018, FruA interacts with other membrane proteins. In this study, the interactions of FruA could be confirmed for GatC, NagE, TreB and MtlA, and the interactions of FruB could be detected and reproduced for MtlA and NagE. The interactions of FruB, the soluble partner, with FruA can be considered as a positive control. Note that the interactions of FruB with MtlA and NagE appear to be substantially weaker than those with FruA.
