# Supplementary material for: Protein:Protein interactions in the cytoplasmic membrane apparently influencing sugar transport and phosphorylation activities of the e. coli phosphotransferase system
Source: PLoS One. 2019 Nov 21;14(11):e0219332. doi: 10.1371/journal.pone.0219332 (PMC6872149; doi:10.1371/journal.pone.0219332)
Supplement: S2 Fig — M, molecular weight markers; Lanes 1 and 2, two purified FruB preparations. (DOCX) [file pone.0219332.s030.docx]

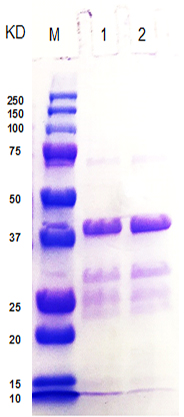


**S2 Fig.** SDS PAGE of the purified FruB preparation from the recombinant *E. coli* BW25113-*fruBKA*:*kn*-pMAL-*fruB* strain. M, molecular weight markers; Lanes 1 and 2, two purified FruB preparations.
