# Supplementary material for: Protein:Protein interactions in the cytoplasmic membrane apparently influencing sugar transport and phosphorylation activities of the e. coli phosphotransferase system
Source: PLoS One. 2019 Nov 21;14(11):e0219332. doi: 10.1371/journal.pone.0219332 (PMC6872149; doi:10.1371/journal.pone.0219332)
Supplement: Attachment — Submitted filename: Review for PlosOne.docx [file pone.0219332.s031.docx]

July 26, 2019

The present manuscript presents experiments that show that modification of protein expression of the fructose-specific phosphotransferase transport (PTS) system has a positive or negative effect on the apparent activity of other sugar-specific PTS systems in *Escherichia coli*. Based on previously published global interactome data, which show that soluble and integral membrane elements of multicomponent PTS systems interact with each other, with the the fructose PTS FruA and FruB components forming the most extensive PTS interactome network, they further analyze the basis of this phenomenon.

They use different physiological conditions related to induction of the fructose specific PTS and different strains of *E. coli,* genetically deleted for FruA or/and FurB, as well as, plasmid-driven overexpression of the Fur components, to investigate how other PTS systems are affected. The authors use basically two functional methods to draw their conclusions; *in vivo* measurements of radiolabeled sugar accumulation in intact cells, and *in vitro* enzymatic assays measuring sugar-specific transphosphorylation apparent activities. They additionally use β-galactosidase transcriptional fusions to test whether the *de novo* synthesis of the affected PTS components is altered in a FurA/B PTS-dependent manner. Based on these assays they conclude that the observed positive or negative effect of FurA/B PTS expression on the activity of other sugar-specific PTS systems take places via protein-protein interaction within the PM, and go on speculating that this is a physiologically meaningful cellular mechanism by which prokaryotes finely regulate PTS-dependent sugar uptake in complex and constantly changing environments.

**Main conclusion**

This is a very interesting scientific story, which happens to be familiar to me, as in my laboratory we have also come across phenomena of dominant negative or positive effects related to apparent transport activities, caused by the overexpression or absence of a specific transporter. Our observations concern fungal transporters, which makes the whole story broader and probably concern the fine and dynamic balancing of PM transporter composition in all kinds of cells. So, the findings described worth publication in PlosOne, but only after some important issues are clarified, conclusion partly modified and some extra experiments are made. In conclusion the text/figures should be partly re-written, conclusions modified, and a western blot analysis performed. Please see details below.

**Points to addressed**

1. The tile of the manuscript is not justified by the results presented. (Protein:Protein Interactions in the Cytoplasmic Membrane Influencing Sugar Transport and Phosphorylation Activities of the E. coli Phosphotransferase System). Protein-protein interactions are apparently the reason of the dominant negative or positive effects observed but this is not formally shown.
2. Introduce a cartoon showing the components of different PTS systems in the introduction. The existing figure is not friendly to the reader. Please use full names when abbreviations appear for first time in the text. What is PEP for example?
3. The first two paragraphs of Results introduction recapitulate published results from the same group. It can be significantly reduced with reference in Table 2 or entirely deleted.
4. *Confirmation of the interactome results using a bacterial two hybrid system*: I cannot see any figure related to this, except S2. It is not clear whether the 2-hybrid system is partly performed here or partly previous publication? Why S2 appears before S1?
5. Having the figure legends interrupting the text in annoying. Either you need both figure/figure legends within the text, or both at the end.
6. In Table S1 explain better what WT and WT-Fr means. I guess the latter means induction by fructose while the former stand for non0induced conditions. One also guess that each experiment is carried out twice, as for each substrate two values are given in all cases, no?  Please, explain abbreviations in all figure legends.
7. Uptakes shown describe ‘apparent’ transporter activity or better ‘apparent accumulation of radiolabelled substrate’, not transport activities per se. Moreover, they are carried out for 5-10 min which definitively describes steady state accumulation and not rate of uptake, despite the results given per min. To my knowledge uptake rates are liner of much shorter times than 5-10 min.,
8. The accumulation of some sugars (e.g. Trehalose and Galactinol) increases significantly (up to 9-fold) in the TM compared to WT upon fructose addition (Table S3). However, the addition of fructose makes a 2-fold difference (Table 2). Please explain this point better in the text.
9. There are some significant deviations in different experiments (S1-S3). Please comment on those.
10. Why S Tables are labelled S4, S5 and S4’, S5’, and not simply with consequent numbers?
11. Most of the data appear in 26 supplementary tables! This makes the manuscript difficult to read and little elegant. I suggest that more clear and composite main figures (with several panels) should highlight the most important results. These should appear in the main text. I am in general against very long supplementary material. The main article should be read without the need for supplements, which are only there to reinforce main findings.
12. A main experiment missing is a western for measuring steady state levels of all components that are affected. Modification of o expression in specific PTS could have an effect on the translocation of PTS in the PM via the translocon complex or in the stability of PTS due to displacement in non-physiological PM microdomains. These possibilities should be discussed.
13. The in vitro PEP-dependent phosphorylation assays are not convincing as they do not lead to a general conclusion. Moreover, I cannot see how crude extracts of membrane proteins can have an effect. My doubts are further enhanced by the fact sugar-P:sugar transphosphorylation assays had no effect by fruA overexpression or purified fruB.
14. “T*he effects appear to be on the transport and PEP-dependent phosphorylation  activities of these enzyme transporters, not on their syntheses or sugar-P:sugar  transphosphorylation activities”* No.  The effect is on apparent activities which is most easily explained by imbalances in protein-protein interactions, including modification in translocation in the PM, stability and/or localization in specific PM microdomains.
15. The first part of the discussion should be deleted, is a repetition of the introduction and results. Also lines 478-507 should be deleted, as they are a repetition of information given ealrler.
