# Supplementary material for: Protein:Protein interactions in the cytoplasmic membrane apparently influencing sugar transport and phosphorylation activities of the e. coli phosphotransferase system
Source: PLoS One. 2019 Nov 21;14(11):e0219332. doi: 10.1371/journal.pone.0219332 (PMC6872149; doi:10.1371/journal.pone.0219332)
Supplement: Attachment — Submitted filename: Response to Reviewers.docx [file pone.0219332.s032.docx]

Dear Dr. Geros,

Thank you for the editorial review of our manuscript, PONE-D-19-16979. We were pleased that you and all three reviewers found merit in the work presented, and concluded that it is “technically complete, well written and relevant.” We also appreciate your suggestion that in general, the reviewers’ concerns can be addressed without further experimentation. We shall include with the revised unmarked manuscript, this rebuttal letter as well as the marked-up copy, highlighting all changes made. It is not important to us whether or not the review history is made public. We have taken measures to ensure that the journal requirements have been satisfied. Our responses to each of the reviewers’ suggestions are indicated below:

**Reviewer #1 (R1)**

We were pleased to read that R1 has observed similar phenomena with fungal transport activities, particularly because there is no PTS in fungi. We have since come to the same conclusion with respect to secondary carriers in *E. coli* (see Discussion section). As noted by R1, “Our observations concern fungal transporters, which makes the whole story broader and probably concern the fine and dynamic balancing of PM transporter composition in all kinds of cells. So the findings described [are] worth publication in PlosOne…”

Points to address:

1. We have inserted the word “Apparently” into the title as suggested. Similar changes have been made throughout the manuscript.

2. We have read through the entire manuscript to ensure that all abbreviations are defined when first used. The scheme shown on pg. 1 of the Introduction has been clarified by additional explanations in the text, both in the Abstract and the Introduction.

3. Table 2 and the first two paragraphs of the Results section summarize results published by Babu et al., 2018 (Ref. 1), as noted by R1, but the relevant observations were not presented concisely or explained in that paper as we have done here for the first time. Therefore, we feel this material should not be omitted from this manuscript. It would be difficult for the reader to find this information in Ref. 1. We also checked for redundancies with the Introduction and feel that most of the information presented is relevant and important; it reinforces the suggestions stated in the Introduction. However, the long section on PTS evolution in the Discussion section that overlapped with the Introduction has been deleted. Thus, although we have eliminated unnecessary details, as well as this major section, we feel that some of this information should be retained.

4. We have included the bacterial 2-hybrid-documented interaction results for FruA and FruB only in Figure S2 because this is only confirmatory of the results already published in Ref. 1 (although using a different method). It is therefore of secondary importance and does not represent novel information; hence we prefer to keep this information in the supplementary materials section.

5. We agree with R1; interrupting text paragraphs is not optimal. In our original submission, we tried to put figures and tables *between* paragraphs, and also tried to keep the tables or figures together with their legends. We have made sure that this was done in the revised manuscript.

6. Yes, R1 is correct: WT-Fru was meant to mean WT grown with Fructose. This has been changed to *WT+Fru* as suggested also by another reviewer. This has also been more clearly explained in the figure legends and text.

7. Uptake or transport activities have been changed to “apparent uptake or transport activities” in relevant places in the manuscript and Tables. Please refer to Aboulwafa et al., 2004 (Arch Microbiol (2004) 181 : 26-34, DOI 10.1007/s00203-003-0623-7) and Aboulwafa and Saier, 2002 (Research in Microbiology 153 (2002) 667-677) to see that apparent transport rate can often be determined within the time range 5-10 min for several of the tested substrates. Please note: “accumulation” is applicable for non-metabolizable compounds only, while rate of uptake plus metabolism is appropriate for other sugars.

8. The high ratios of accumulation of Tre and Gat by TM/WT is due to the lower activities of the WT after growth in the presence of fructose, not to a change in uptake by the TM. The results are fully consistent with our interpretation of the data.

R1 notes an apparent discrepancy between Tables S3 and 2. I assume R1 means Table S2 instead of Table 2? Table 2 only presents relevant interaction scores from Ref. 1.

In Table S2, only the TM strain is examined, and with this strain, there is no fructose PTS; hence, fructose has no effect on uptake. However, in Table S3, WT uptake activity is low, compared to the TM because of the inhibition of Gat and Tre uptakes by II^Fru^, and hence the ratio of TM+Fru (no effect)/WT+Fru (strong inhibition) is large. This is because only the fructose-induced WT shows inhibition by high levels of FruA on Gat and Tre uptakes. These experiments also establish that FruAB is responsible for the activation/inhibition of the other PTS transporters.

9. The reviewer is correct; there are significant deviations between experiments compared to the standard deviations for any one experiment conducted with the same cells, but we still attribute these to experimental error. This has been noted in the text.

10. As noted above, the S’ tables are controls for the corresponding S tables.

11. The supplementary tables provide the raw data upon which the figures and tables in the main manuscript are based. The relevant S tables for each text table are given in the text table legends. To include S tables in the text would introduce substantial redundancy and could confuse the reader as this would introduce a different style of presentation.

12. These possibilities are now discussed in the Discussion section as suggested.

13. We believe that the PEP-dependent *in vitro* phosphorylation results are significant. They confirm previous studies showing that cell disruption seems to (at least partially) uncouple sugar transport from phosphorylation. Values reported are much greater than the standard error of the measurements, and they show that in some cases, the *in vitro* effects observed are in the same direction as the *in vivo* effects although smaller in magnitude, possibly because cell disruption also disrupts protein-protein interactions. This has also been considered in the Discussion section of the revised manuscript.

14. We have qualified this statement (which we still believe to be essentially true) by mentioning the possibility of an effect on stability of the target transporters, although as noted above, and now stated in the revised manuscript, this seems less likely. Thus, the transphosphorylation reactions were not affected, suggesting that the target EIIs are stable. Moreover, it is known that EIIs are very stable in cell membranes (for days at RT and up to ~80^o^C for short time periods). This argument and these facts have now been presented in the revised text.

15. It is true that the first two paragraphs in the Discussion section in part paraphrase parts of the Introduction, but we believe this helps the reader to view the results in a fully comprehensive picture. We therefore prefer to retain this information. However, if acceptance for publication depends on its removal, we will certainly do so. Also, we have substantially shortened the Discussion section concerning PTS evolution because of the overlap with the Introduction.

**Reviewer #2 (R2)**

1. When we discuss mannose transport, we are talking about the results obtained with the non-metabolizable mannose analog, 2DG, which is a good substrate only of the mannose system. This point is now clarified in the revised manuscript (in lines 251 and 280 of the original manuscript) by noting that 2DG accumulation reflects the activity of the mannose PTS. This has also been clarified by putting the primary substrates into Table 1, as suggested.

2. Yes, we agree, and this has been done.

3. This is an interesting point. We believe that while induction by including fructose in the growth medium led to a full complement of regulatory effects, artificial overproduction of proteins from the gene(s) encoded on a synthetic plasmid did not. It caused the activating effects but did not reproduce the inhibitory effects on the Gat and Tre systems. We assume that this latter observation was due to the abnormal condition resulting from the presence of an overexpression plasmid, from which transcription/translation must occur. Possibly, these conditions (partially) prevent formation of the necessary association between FruA/FruB and the inhibited systems. This is mere speculation, but we have mentioned it in the Results section. This suggestion is also supported by studies on adenylate cyclase activation by the PTS which is easily demonstrable in whole cells and even toluenized cells, but never in a fully *in vitro* assay system.

4. Lines 350-351: We believe this statement is justified because the homologous HPr and FPr catalyze the same reaction, using the same mechanism, with the same specificity. If FPr were responsible for the activation, an excess of HPr should have had the same activating effect, but it did not. It had little or none; this is now more clearly pointed out.

5. Line 372, *galP*: a brief description of GalP has been added (as suggested) to the figure 2 legend.

6. Lines 41-42: R2 is correct, that we have only shown that synthesis (gene expression) is not affected, but turnover was not tested rigorously. This has been noted in the text as mentioned above and the statement in the abstract has also been accordingly modified. However, please note that the transphosphorylation results and other evidence now cited in the manuscript argue against the possibility that turnover plays a role.

**Reviewer #3 (R3)**

We thank R3 for the positive comments, particularly: “The authors of this article provide novel insights into a new mode of control” and “The experiments fully support the conclusions…”

Minor concerns:

Line 41: Thank you for catching this Typo! It has been corrected.

Line 58: As noted above, we have provided substantially more description in the text regarding this scheme and feel that these additions render it much more easily understandable. Similar schemes have been published in numerous publications concerning the PTS in the past.

Line 189/Table 2: This was addressed above. Since these results merely confirm the previously published results (Babu et al., 2017; Ref. 1) and do not represent an important conclusion with respect to the substance of this paper, we believe this figure optimally belongs in supplementary materials. The results now appear in S2 Figure.

Tables S1 and S2: This has also been addressed above. Please note that different strains and conditions were used for these and Tables S3 etc., accounting for the differences, excluding experimental error.

Tables S1-S5: We agree and have changed WT-Fru or WT-Fr to “WT + Fru”. The same has been done for other tables.

Figure S2 (pg. 31): The evidence for several relevant interactions is presented.

We are grateful to you and the reviewers for the useful editorial review and hope the changes and corrections made in response to the Reviewers’ suggestions render the manuscript acceptable for publication in PLOS ONE.

Sincerely yours,

Milton Saier
